# Building HMM and molecular docking analysis for the sensitive detection of anti-viral pneumonia antimicrobial peptides (AMPs)

**DOI:** 10.1038/s41598-021-00223-8

**Published:** 2021-10-18

**Authors:** Olalekan Olanrewaju Bakare, Marshall Keyster, Ashley Pretorius

**Affiliations:** 1 Bioinformatics Research Group, University of the Western Cape, Cape Town, 7535 South Africa; 2 Environmental Biotechnology Laboratory, Biotechnology Department, University of the Western Cape, Cape Town, 7535 South Africa

**Keywords:** Biotechnology, Computational biology and bioinformatics, Biomarkers

## Abstract

Pneumonia is the main reason for mortality among children under five years, causing 1.6 million deaths every year; late research has exhibited that mortality is increasing in the elderly. A few biomarkers used for its diagnosis need specificity and precision, as they are related to different infections, for example, pulmonary tuberculosis and Human Immunodeficiency Virus. There is a quest for new biomarkers worldwide to diagnose the disease to defeat these previously mentioned constraints. Antimicrobial peptides (AMPs) are promising indicative specialists against infection. This research work used AMPs as biomarkers to detect viral pneumonia pathogens, for example, Respiratory syncytial virus, Influenza A and B viruses utilizing in silico technologies, such as Hidden Markov Model (HMMER). HMMER was used to distinguish putative anti-viral pneumonia AMPs against the recognized receptor proteins of Respiratory syncytial virus, Influenza A, and B viruses. The physicochemical parameters of these putative AMPs were analyzed, and their 3-D structures were determined utilizing I-TASSER. Molecular docking interaction of these AMPs against the recognized viral pneumonia proteins was carried out using the PATCHDOCK and HDock servers. The results demonstrated 27 anti-viral AMPs ranked based on their E values with significant physicochemical parameters in similarity with known experimentally approved AMPs. The AMPs additionally had a high anticipated binding potential to the pneumonia receptors of these microorganisms sensitively. The tendency of the putative anti-viral AMPs to bind pneumonia proteins showed that they would be promising applicant biomarkers to identify these viral microorganisms in the point-of-care (POC) pneumonia diagnostics. The high precision observed for the AMPs legitimizes HMM’s utilization in the disease diagnostics’ discovery process.

## Introduction

The high mortality emerging from pneumonia infection requires the hunt for new diagnostic strategies and biomarkers to determine patients’ status before the onset of signs and symptoms^1^. Mortality from pneumonia is partly due to delayed therapy because there is no sensitive approach to identifying the disease’s viral causation. A few biomarkers that, until now, have been utilized in the detection of the sickness are unpredictable as they are involved in different illnesses, consequently giving false-positive or false-negative results^2^. Antibodies that have been said to be the best quality standard for disease detection because of their high sensitivity have, as of late, been connected to different weaknesses, for example, cross-reactivity^3^. The problems mentioned above contribute to the poor diagnosis of the disease resulting in drug abuse and drug resistance by microorganisms. Henceforth the need to make a quest for sensitive biomarkers for pneumonia diagnosis is essential.

Several diagnostic biomarkers have been connected to pneumonia, among which are C-reactive protein (CRP), Procalcitonin (PCT), a Soluble triggering receptor expressed on myeloid cells-1 (STREM-1), CD163, and High Mobility Group Box-1(HMGB-1). It is known that CRP and PCT have proven valuable in the detection of the disease as they are created in an impressively high amount. Yet, there is uncertainty in their sensitivity towards pneumonia as they can be produced as a result of other inflammatory stimuli in the neuron, atherosclerotic plaques, myocytes, and lymphocytes^4^; while the mechanism controlling their production at these locales is not established^5^. There is ongoing research into discovering more sensitive biomarkers for pneumonia diagnosis to solve its diagnosis and treatment^6^ completely. These include the involvement of other biomarkers for their likely utilization in pneumonia diagnosis.

Additionally, several methods exist to detect pneumonia pathogens, ranging from blood cultures, polymerase chain reaction, matrix-assisted laser desorption or ionization-time of flight, immunofiltration, turbidimetric immunoassay dependent on latex agglutination, to mention a few^1^. It is possible to detect locally confined bacteria/viruses causing pneumonia. However, these techniques endure inadequacies, for example, the lack of sensitivity of blood cultures^6^, the failure of X-ray to distinguish the causative microorganism^7^, absence of precision of the polymerase chain reaction^8^, failure to detect viral pneumonia in the matrix-assisted laser desorption or ionization-time of flight^9^, high cost and absence of sensitivity of immunofiltration and turbidimetric immunoassay^10^. Thus, there is a need to discover new methods or improve the existing ones for sensitive and accurate disease detection to eliminate false-positive/false-negative results.

Antimicrobial peptides (AMPs) are small molecular weight oligopeptides with an expansive range of antimicrobial activity against bacteria, viruses, and fungi. These peptides have been conserved throughout evolution with hydrophobic and hydrophilic side chains that help them traverse the aqueous environment and lipid-rich biological membranes^11^. Likewise, recent research has demonstrated that AMPs enhance the host immunity through receptor-dependent interactions, which have significance in different capacities such as angiogenesis, wound healing, and chemotaxis^12^. The recent roles of the AMPs suggest that they are significant and already undervalued molecules. In work by Tincho, Gabere (13), for instance, a list of experimentally approved anti-HIV AMPs was discovered utilizing HMM to develop a few sensitive models for their prediction. This research established the discovery of a few AMPs that bound the HIV p24 protein for the sensitive diagnosis of both HIV 1 and 2 through the construction of a lateral flow device (LFD)^14^.

Many in silico tools exist to distinguish AMPs, among which is HMMER uses a profile strategy for AMPs prediction. Each sequence is exhibited as a bunch of similitudes (probabilities) with a group of successions models^15^. The development of a profile is typically restricted to the utilization of positive information (functional sequences) without discrimination. HMM is used to compute statistical analysis of different DNA alignments, to distinguish genomic features, for example, insertions, deletions, substitutions, and to identify protein domains for homology modeling of protein families^16^. Clusters by HMM likewise permit a minimum amount of likeness between all peptides. Another significant element of HMMER is the capacity to capture data by preserving the content in a sequence alignment^17^. For this reason, high molecular structures, for example, protein domains, are regularly characterized utilizing HMMER^18^. This research aims to discover novel AMPs as biomarkers to recognize viral pneumonia due to the high mortality related to the illness with the aid of in silico tools, such as HMMER, to accelerate the discovery process.

## Materials and methods

### Data retrieval (literature mining)

The experimentally approved anti-pneumonia AMPs for the viral pathogens (Respiratory syncytial virus, Influenza A and B viruses) were recovered from the antimicrobial peptide databases, for example, Antimicrobial Peptides Database (APD3)^19,20^, Collection of Antimicrobial Peptides (CAMP)^21^, and Anti-viral peptides databases (AVPDB)^22^. Curation was carried out through literature mining to affirm that all the recovered AMPs were either experimentally approved or anticipated. Duplicate experimentally validated AMPs were then removed from the recovered list utilizing the Cluster Database at High Identity with Tolerance (CD-HIT)^23^.

### Training and testing datasets (data mining)

The final list of the experimentally validated AMPs was sorted by their particular pathogenic strains with INFA—anti-Influenza A; INFB—anti-Influenza B; and RSV-anti-Respiratory Syncytial Virus^24–26^. Every classification of the strain-specific datasets was arbitrarily separated into two subsets: seventy-five percent of every dataset was used as the training set (to assemble each profile). At the same time, one-quarter was utilized as the testing dataset.

### Construction of AMPs profiles (text mining)

The HMMER algorithm version 2.3.2^27^ was utilized to build detailed pathogen-targeted models/profiles utilizing the training datasets. All the HMM profiles were constructed on the Ubuntu 12.04 LTS operating system. The assignment was cultivated on a terminal, and the command lines used to fabricate each profile was composed by the corresponding algorithm and the means associated with their development were as beneath:For the initial step, the training datasets of each target class were adjusted utilizing the ClustalW alignment device^28^. The task was carried out utilizing the command line:
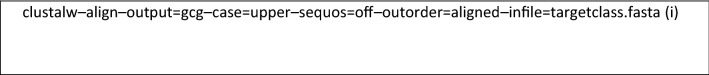


The command line essentially states ≪do an alignment of the sequences which are in the capitalized form found in the input record “target class.fasta” with the FastA, utilizing ClustalW as numerous alignment instruments and GCG Postscript yield for graphical printing≫. The command’s yield brings about the development of adjusted sequences, called “target class.msf”. The modified sequences were utilized as a contribution to the subsequent stage.

The subsequent stage upgrades the development of the profiles of the target class sequences by indicating the normal motifs/signatures inside the profiles. To accomplish this, the “Build profiles” was run utilizing the accompanying command line:
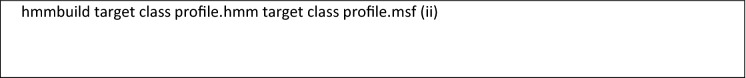


To improve the sensitivity of the profiles, the document created (target class. hmm) from the profile building step was constructed by utilizing the order line:
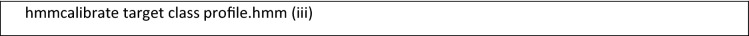


The subsequent profiles “target class.hmm” was utilized in assessing the profiles execution by testing the built profiles on a free AMP dataset.

### Independent profile testing

The independent testing of each constructed profile was carried out in a stage called “Query profiles” The testing datasets were queried against the constructed profiles utilizing the command line, with an E-value of 0.05:
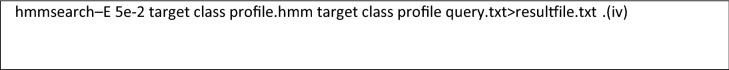


### Performance measurement of each profile

Statistical performance measures were then determined utilizing sensitivity, specificity, accuracy, and Matthews Correlation Coefficient (MCC) as indicators. The measures utilized are depicted as follows: Sensitivity is the percentage of anti-pneumonia AMPs against a specific microbe (testing sets) accurately anticipated as anti-pneumonia AMPs (positive). The sensitivity is characterized by the Eq. (1):

1 $${\text{Sensitivity}} = \left( {\frac{TP}{{TP + FN}}} \right) \times 100$$

Specificity is the level of non-anti-pneumonia AMPs (negative datasets) effectively anticipated as non-anti-pneumonia AMPs (negative). The specificity is characterized in Eq. (2):

2 $${\text{Specificity}} = \left( {\frac{TN}{{TN + FP}}} \right) \times 100$$

Accuracy is the percentage of accurately anticipated peptides (anti-pneumonia AMPs and non-anti-pneumonia AMPs). The accuracy is characterized in Eq. (3):

3 $${\text{Accuracy}} = \left( {\frac{TP + TN}{{TP + FP + TN + FN}}} \right) \times 100$$

Matthews correlation coefficient (MCC) is a proportion of both sensitivity and specificity. MCC = 0 shows arbitrary expectation, while MCC = 1 demonstrates the perfect forecast. It is characterized in Eq. (4):

4 $${\text{MCC}} = \left( {\frac{(TP \times TN) - (FN \times FP)}{{\sqrt {(TP + FN) \times (TN + FP) \times (TP + FP) \times (TN + FN)} }}} \right)$$

### Identification of novel putative anti-Pneumonia AMPs from proteome sequences

Proteome sequences were queried by the profiles with the list of all proteome sequences (in the fasta design) recovered from the Ensembl information base (http://www.ensembl.org/index.html) and the UniProt information base (http://www.uniprot.org/). A cut-off E-value was set to be 0.05 for the retrieval of putative anti-pneumonia AMPs. This was cultivated utilizing “hmmsearch” module of the HMMER software with the command line utilized expressed underneath: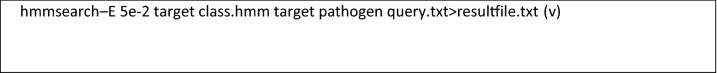 where the target class.hmm in one of the three profiles, target class query.txt speaking to the species examined against the profile and result file.txt is the outcome document realized from querying the species against a specific microbe profile.

### Identification of receptors

Viral receptors, for example, cell surface receptors and nucleoproteins, were recognized for the viral causative pathogens (Respiratory syncytial virus, Influenza A, and B) involved in pneumonia to fill in as targets for the distinguished AMPs utilizing a few *in-silico* strategies. Viral pneumonia proteins were gathered from different protein data banks (PDB), for example, the National Center for Biotechnology Information (NCBI), UniProt, Google Scholar, and Ensembl through literature mining. Curation was performed to confirm that all the recovered viral pneumonia proteins were complete or incomplete. Fractional proteins were removed, and complete protein was retained for additional examination. BLAST investigation was performed utilizing the UniProt interface for further affirmation of specificity with the end goal that the viral pneumonia proteins retrieved were absent in other microorganisms and viruses.

### Physicochemical properties of the putative anti-Pneumonia AMPs and the pneumonia proteins

Physicochemical properties of the putative anti-pneumonia AMPs and pneumonia receptor proteins were determined utilizing the calculation interface of Bactibase (http://bactibase.pfba-lab-tun.org/physicochem)^28,29^ and APD3 (https://wangapd3.com/main.php)^18,19^ utilizing the amino acid sequences of the putative peptides as information.

### De novo structure predictions of the putative anti-Pneumonia AMPs and Pneumonia proteins (receptors) using I-TASSER

3-D structures of the anti-Pneumonia AMPs and the viral pneumonia receptor proteins were predicted by transferring each sequence onto the I-TASSER (Iterative Threading ASSEmbly Refinement) site^29^. The 3-D structures of the AMPs and their protein receptors were visualized utilizing the PyMOL version 1.3^30^. This was accomplished by downloading the most recent version of the PyMol on Ubuntu Linux, utilizing the terminal command line.

### Docking analysis of the putative anti-pneumonia AMPs and Pneumonia Proteins 3-D structures using PatchDock

The 3-D structures of the anti-viral Pneumonia putative AMPs and the viral pneumonia protein receptors PDB files from I-TASSER were transferred onto the PatchDock server using the default RMSD of 4 and subsequent selection of “protein-small ligand”^31^. HDock, an efficient molecular docking algorithm with an accurate scoring function for biomolecular interactions, was also used as an alternative docking tool to check the consistency of the PatchDock tool^32^. This tool combined Physics with Bioinformatics-based methods to generate structure prediction and interaction.

Interaction analysis between the anti-viral Pneumonia putative AMPs and their respective viral pneumonia protein receptors was carried out utilizing the PyMol software^30^.

## Results

### Retrieval of anti-viral AMPs (VAP-AMPs) and profile creation using HMM

Literature mining uncovered 176 experimentally validated anti-viral pneumonia antimicrobial peptides (VAP-AMPs) in total for the CAMP, APD3, and AVPDB databases against the microbes Respiratory Syncytial Virus, Influenza A, and B in the order 112, 52 and 12, respectively. The initial phase in the profile construction pipeline was the random grouping of the various classes into ¾ and ¼ of the experimentally validated AMPs (Table 1). The ¾ is the training dataset, expected to prepare the HMM software to test whether the functionally significant amino acid consensus is captured. After this, multiple alignments were produced utilizing HMM ClustalW. A total of three AMP profiles was produced for every one of the accompanying classes ((anti-Respiratory syncytial virus (RSVM) and, anti-Influenza A, and B (INFA and INFB).

**Table 1:** Profile creation by HMM.

Profiles	Training datasets	Testing datasets	Total
INFB	9	3	12
INFA	39	13	52
RSVM	84	28	112

*INFA* anti-Influenza A virus, *INFB* anti-Influenza B virus; *RSVM* anti-respiratory syncytial virus.

### Independent testing of the created profiles and evaluation of the independent testing results

Each constructed profile was tested against a positive dataset (testing datasets) which was about 25% of the dataset (training datasets in Table 1). Since experimentally validated AMPs were used, the assumption is that the profiles developed ought to have the option to recognize different sequences with precisely the same action and separate those that have no anti-pneumonia activity from the same microorganism. The constructed profiles were examined against a negative control dataset, comprised of random fragments of 17,236 neuropeptides, which had no recorded anti-pneumonia action. This independent testing was carried out with the negative dataset (neuropeptides) to confirm whether the trained profiles would distinguish non-anti-pneumonia peptides.

The independent testing of the profiles was evaluated utilizing the true positive (TP), false-positive (FP), true negative (TN), and false-negative (FN). A cut-off E-value of 0.05 was applied to the HMM tool to fortify the profile’s capacity to separate between the TP anti-pneumonia AMP and the false-negative anti-pneumonia AMPs. TP speaks to effectively anticipated positive sequences (anti-pneumonia AMPs), TN indicates accurately predicted negative groupings (non-anti-pneumonia AMPs), FP (False-positive) is the quantity of non-anti-pneumonia AMPs wrongly anticipated as anti-pneumonia AMPs (AP-AMPs), FN is the number of anti-pneumonia AMPs wrongly anticipated as non-anti-pneumonia AMPs. It was conceivable to ascertain the quantity of TP AMPs from the complete number of input sequences; accordingly, the FP number could be extrapolated with the outcomes that appeared in Table 2, mirroring the limit of each profile to recognize true anti-pneumonia AMPs from false anti-pneumonia AMPs. In Table 2, INFB had all its testing datasets as TP while RSVM had 22 of its 28 testing datasets as TP. Nonetheless, INFA had 6 of its 13 testing datasets as TP, which could be because of an overlap of homologous relationships in the AMPs utilized in their profiles.

**Table 2:** Independent testing of profiles against test and negative datasets.

Profiles	TP	FN	TN	FP
INFB	3	0	17,236	0
INFA	6	7	17,236	0
RSVM	22	6	17,236	0

*INFA* anti-Influenza A virus, *INFB* anti-Influenza B virus, *RSVM* anti-Respiratory Syncytial Virus, *TP* true positive, *TN* true negative, *FP* false-positive, *FN* false-negative.

### Performance measurement of the target-specific profiles

After evaluating the capacity of the tested profiles, the performance was determined to calculate the performance of each profile, utilizing specificity, sensitivity, accuracy, and MCC, presented by organic chemist Brian W. Matthews in 1975^33^. The specificity, sensitivity, accuracy, and MCC were determined as detailed in Table 3.

**Table 3:** Summary of performance measurement of the profiles.

Models	Sensitivity (%)	Specificity (%)	Accuracy (%)	MCC
INFB	100	100	100	1
INFA	46	100	99.1	0.68
RSVM	79	100	99.96	0.89

*INFA* anti-Influenza A virus, *INFB* anti-Influenza B virus, *RSVM* anti-Respiratory syncytial virus,* MCC* Matthews correlation coefficient.

From the results in Table 3, sensitivity values were high in Anti-*Influenza B virus* (INFB) and Anti-*Respiratory Syncytial Virus* (RSVM) of anti-viral profiles tested. The high sensitivity values of INFB and RSVM profiles indicated the right prediction. The moderate sensitivity of INFA could be ascribed to the huge overlap in the conserved space of the AMPs utilized for their profile development^17^. The specificity results for all profiles were 100%, indicating a correct prediction. The accuracy results of the profiles showed a correct prediction with the elimination of mistakes by invalidating misclassified AMPs from both positive and negative datasets. MCC values for all the profiles indicated huge outcomes, with the most minimal value recorded for Anti-*Influenza A virus* (INFA) (0.50). The MCC value of 0.5 to 1 relates to the ideal expectation, while ‘0’ points to an irregular prediction. Hence all profiles showed right expectation (INFB > RSVM > INFA). The MCC is considered to give the best performance estimation of models since it joins sensitivity, specificity, and accuracy^33^.

### Proteome sequence databases query and discovery of putative anti-pneumonia AMPs

The discovery stage (Table 4) was to look for novel anti-viral pneumonia AMPs for the pneumonia pathogens (Influenza A, B just as Respiratory Syncytial Virus) in order to recognize peptides that had similar signatures/motifs and properties as the input sequences used to assemble the profiles RSVM, INFA, and INFB. The matches of the separate profiles to the proteome sequences additionally appeared with E-values (Table 4) of 0.05 to discover putative AMPs. The final list of anti-viral AMPs was arranged by their E-values, with those having the smallest E-values described as the most probable putative anti-viral pneumonia AMPs.

**Table 4:** Final list of the Anti-bacteria and Anti-viral pneumonia AMPs with their sources.

S/N	TARGET	ORGANISMS	E-VALUE	SEQUENCE
1	BOPAM-INFA1	*Ciona savignyi*	0.015	TPTFI----PIPKQ
2	BOPAM-INFA 2	*Nematostella vectensis*	0.034	CPVIL----QVLPK
3	BOPAM-INFA 3	*Ciona savignyi*	0.035	TPTF----VTVPDQ
4	BOPAM-INFA 4	*Ciona savignyi*	0.036	TPTF----VPMPQQ
5	BOPAM-INFA 5	*Ciona savignyi*	0.037	TPTF----VPIPQQ
6	BOPAM-INFA 6	*Ciona savignyi*	0.038	TPKF----VPIPEQ
7	BOPAM-INFA 7	*Nematostella vectensis*	0.039	CPVI----IQVFPK
8	BOPAM-INFA 8	*Ciona savignyi*	0.040	TPTF----VPIPEQ
9	BOPAM-INFB1	*Callithrix jacchus*	0.0045	MDV----TFLRVPPQ
10	BOPAM-INFB2	*Callithrix jacchus*	0.0055	MDV----TFLMVPPQ
11	BOPAM-INFB3	*Xenopus amphibian query*	0.0057	LNC----LCLNCNPQ
12	BOPAM-INFB4	*Vicugna pacos*	0.0059	LKI----LFLKVPQL
13	BOPAM-INFB5	*Xenopus amphibian query*	0.0060	LTI----LFLKVPQL
14	BOPAM-INFB6	*Xenopus amphibian query*	0.0064	LNC----LCLNCNPQ
15	BOPAM-RSV1	*Brachyspira murdochii*	0.0057	IVSS----INLCKNKF
16	BOPAM-RSV2	*Brachyspira hyodysenteriae*	0.0074	EVSK----IDSNLSTV
17	BOPAM-RSV3	*Veillonella atypica*	0.0076	NIVD----IDANTTAI
18	BOPAM-RSV4	*Veillonella atypica*	0.0077	KVTE----IDNNVNII
19	BOPAM-RSV5	*Veillonella atypica*	0.0079	AINS----VNKNTNNI
20	BOPAM-RSV6	*Bartonella grahamii*	0.0080	KFSQ----IDMQTSVI
21	BOPAM-RSV7	*Veillonella dispar*	0.0082	NISN----LNQNINNV
22	BOPAM-RSV8	*Bartonella grahamii*	0.0084	SIYN----SNVLLSAV
23	BOPAM-RSV9	*Veillonella dispar*	0.0087	NISK----VNTNTTNI
24	BOPAM-RSV10	*Veillonella dispar*	0.0088	VIND----VNTNTTNI
25	BOPAM-RSV11	*Veillonella dispar*	0.0089	NVTN----VDVNTADI
26	BOPAM-RSV12	*Veillonella dispar*	0.0091	DITNI----DAKFTKI
27	BOPAM-RSV13	*Brachyspira hyodysenteriae*	0.0094	KIRD----LDDRITNV

*BOPAM-INFA1-13* putative anti-Influenza A virus AMP, *BOPAM-INFB1-7* putative anti-Influenza B virus AMP, *BOPAM-RSV1-13* putative anti-Respiratory Syncytial Virus AMP.

“-” represents the coded amino acid residues that are available subject to request.

### Physicochemical properties of the AMPs

The physicochemical parameters of the putative AMPs were determined using APD3 and BACTIBASE to ascertain that the AMP sequences conform to other known AMPs. Physicochemical parameters, for example, atomic weight amino acid components, hydrophobicity, Boman index, net charge, isoelectric potential, and half-life, were utilized to assess the anti-viral AMPs (Table 5). The amino acid composition of the AMPs adds to the molecular weight since the AMPs are comprised of amino acids and can be a distinctive component to separate between two classes of protein/eptides^34^. Aside from this, the anti-viral pneumonia AMPs likewise have common amino acids that could recognize them from each other. BOPAM-INFA1, 6, and 8 had proline; BOPAM-INFA2 had proline, valine, isoleucine, and leucine; BOPAM-INFA3 had threonine; BOPAM-INFA4-5 had proline and glutamine; while BOPAM-INFA7 had proline, isoleucine, serine, and valine. BOPAM-INFB1 had valine and proline; BOPAM-INFB2-3 had asparagine and leucine; BOPAM-INFB4-5 had leucine; while BOPAM-INFB6 had asparagine and leucine. BOPAM-RSV1 had isoleucine and lysine; BOPAM-RSV2 had serine; BOPAM-RSV3 and 12 had isoleucine and asparagine; BOPAM-RSV4, 5, 7, 8, 9, 10, 11 had asparagine; BOPAM-RSV6 had isoleucine while BOPAM-RSV13 had aspartate. The anti-pneumonia AMPs such as BOPAM-INFA1, 3, 4, 5, 6, and 8, BOPAM-RSV9 had hydrophobicity less than 30% due to the presence of more polar amino acid residues. All the anti-viral peptides such as BOPAM-INFB3, 6, BOPAM-INFA1, 2, 3, 4, 6, 7, 8, BOPAM-RSV2, 3, 4, 6, 7, 8, 10, 11, 12, 13 were predominantly neutral or negative. Cationic AMPs are said to be positively connected with expanded antimicrobial activities^35^. Nonetheless, the absence of the positive charge in the net charge of anti-viral AMPs does not interpret an absence of antimicrobial activities since some negatively charged AMPs have recently been discovered, for example, surfactant associated anionic peptides in the APD3 database (AP00528) with a net charge of − 5 which has antibacterial activity and maximin H5 with charge ranging between − 1 and − 7 which has bacterial growth inhibition against *Listeria monocytogenes*^33^. Anti-viral pneumonia AMPs pI range from 3.85 to 12.50 shows solubility properties for the AMPs regardless of the difference in charges of acid and alkaline media^36^. Isoelectric potential (pI) of peptides is an element of individual amino acids in the backbone groups. At a pH beneath the pI, AMPs convey a net positive charge and vice versa. The outcomes of the Boman index demonstrated negative values for BOPAM-INFA2, 7, and BOPAM-INFB4, and 5. A negative Boman index is said to be positively correlated with a more hydrophobic peptide, showing a high protein binding potential, while a more hydrophilic peptide will, in general, have a more positive index^37^. Notwithstanding, specific peptides’ inclination to have a positive Boman index has been accounted for with the capacity to distinguish HIV in a lateral flow device^13^. Anti-viral pneumonia AMPs had BOPAM-INFA1, 3, 4, 5, 6, 8 with a half-life of 7.2 h, BOPAM-INFA2, and 7 had a half-life of 1.2. BOPAM-INFB1, 2 had a half-life of 30 h, BOPAM-INFB3-6 had a half-life of 5.5 h. BOPAM-RSV1 had a half-life of 20 h, and BOPAM-RSV10 had a half-life of 100 h; all other BOPAM-RSV had a half-life range between 1 and 4.5 h. AMPs have been said to generally exhibit a short half-life because they are not stable. Half-life values as low as 1 h have been reported for AMP molecules used for HIV diagnosis^13^.

**Table 5:** Physicochemical parameter of the antibacterial and antiviral pneumonia putative AMPs.

S/N	AMPs	Mass number(Da)	% Hydrophobic	Common amino acid	Net charge	PI	Boman Index (kcal/mol)	Half-life (h)
1	BOPAM-INFA1	1 541.41	28	P	0	6.34	0.9	7.2
2	BOPAM-INFA2	1 496.20	57	PVIL	0	0.16	− 0.54	1.2
3	BOPAM-INFA3	1 518.28	28	T	− 2	3.49	1.37	7.2
4	BOPAM-INFA4	1 559.68	28	QP	− 1	7.52	3.75	7.2
5	BOPAM-INFA5	1 541.64	28	PQ	− 1	3.75	0.9	7.2
6	BOPAM-INFA6	1 569.42	28	P	− 1	4.18	1.2	7.2
7	BOPAM-INFA7	1 546.21	50	PISV	0	6.16	− 0.03	1.2
8	BOPAM-INFA8	1 542.35	28	P	− 2	3.55	0.99	7.2
9	BOPAM-INFB1	1 869.55	40	VPR	+ 2	10.40	2.27	30
10	BOPAM-INFB2	1 844.56	46	NL	+ 1	7.55	1.12	30
11	BOPAM-INFB3	1 687.62	46	NL	0	5.76	0.94	5.5
12	BOPAM-INFB4	1 780.00	66	L	+ 2	10.81	− 1.61	5.5
13	BOPAM-INFB5	1 752.93	66	L	+ 1	9.70	− 1.81	5.5
14	BOPAM-INFB6	1 656.33	46	NL	0	5.76	0.57	5.5
15	BOPAM-RSV1	1 865.32	43	IK	+ 1	8.54	1.39	20
16	BOPAM-RSV2	1 776.07	31	S	− 1	4.43	2.29	1
17	BOPAM-RSV3	1 729.06	43	IN	0	6.45	1.69	1.4
18	BOPAM-RSV4	1 784.11	43	N	− 1	4.18	1.7	1.3
19	BOPAM-RSV5	1 687.21	37	N	+ 1	9.70	2	4.4
20	BOPAM-RSV6	1 813.76	50	I	− 1	4.18	0.89	1.3
21	BOPAM-RSV7	1 827.37	31	N	0	6.41	2.8	1.4
22	BOPAM-RSV8	1 784.05	43	N	− 1	3.85	1	1.9
23	BOPAM-RSV9	1 761.30	25	N	+ 1	9.7	2.5	1.4
24	BOPAM-RSV10	1 760.31	31	N	0	6.34	2.21	100
25	BOPAM-RSV11	1 716.96	37	N	− 1	4.11	2.14	1.4
26	BOPAM-RSV12	1 834.16	37	IN	0	6.45	2.23	1.4
27	BOPAM-RSV13	1 942.32	31	D	0	6.60	4.12	1.3

*S/N* serial number, *BOPAM-INFA1-13* putative anti-Influenza A virus AMP, *BOPAM-INFB1-7* putative anti-Influenza B virus AMP, *BOPAM-RSV1-13* putative anti-Respiratory Syncytial Virus AMP, *PI* represents the isoelectric point of the AMPs.

### Retrieval of protein receptors of pneumonia pathogens

This stage was carried out‏ to assess the diagnostic potential of some immunogenic proteins of viral pneumonia to serve as targets for the putative antimicrobial peptides to determine these microbes. For example, a few pneumonia proteins, cell surface receptors, and nucleoproteins were analyzed for the viruses: Influenza A, Influenza B viruses, Respiratory Syncytial virus. These recovered protein receptors were projected to be potentially applicable in the diagnosis of viral pneumonia associated with these viruses. The Respiratory syncytial virus has some immunogenic receptors that have potential diagnostic pertinence, such as membrane fusion core protein chains^38^. The virus has Human RSV fusion protein core chain A with molecular weight 4869.38 Da, isoelectric point 4.38, hydrophobicity 39.53%, charge—4, instability index 49.41, and half-life of 1 h in mammals. Influenza A virus has some protein receptors of potential importance in its detection. It has 416a monomeric nucleoprotein with molecular weight 56,297.78 Da, isoelectric point 9.45, hydrophobicity 29.52%, charge + 12, instability index 36.35, and half-life of 30 h in mammals. Influenza B virus receptor proteins of diagnostic potential were recognized and investigated. Influenza B virus has nucleoprotein with molecular weight 61,644.09 Da, isoelectric point 9.43, hydrophobicity 31.61%, charge + 18, instability index 39.98 half-lives of 30 h in mammals (Table 6). Instability index, molecular weight, and half-life are a function of how stable a protein can be, and any protein with an instability index lower than or equal to 40 is said to be stable; hydrophobicity enhances protein binding to ligands; while the net charge determines the behavior of the proteins in acidic or alkaline solution with all proteins having a net zero charge at the isoelectric point^39^.

**Table 6:** Physicochemical properties of the retrieved pneumonia receptor proteins.

S/N	Anti-Pneumonia proteins	Molecular weight (Da)	Hydrophobicity (%)	Net charge	Instability index	Half-life (hours)
1	INFA R416a monomeric Nucleoprotein	56,297.78	29.52	+ 12	36.35	30
2	INFB Nucleoprotein	61,644.09	31.61	+ 18	39.98	30
3	RSV Chain A Matrix protein	28,990.34	35.66	+ 3	33.15	1

*INFA* Influenza A virus, INFB- Influenza B virus, *RSV* Respiratory Syncytial virus.

### Structure prediction of the putative anti-pneumonia AMPs and Pneumonia protein receptors

**Figure 1:**
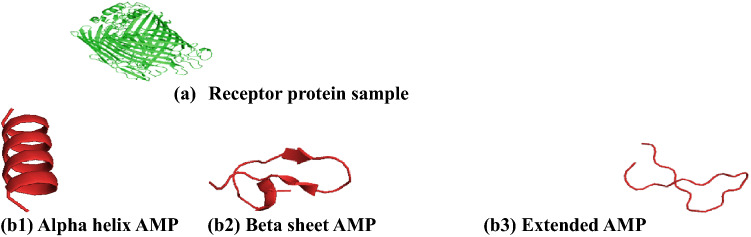
3-D structures of the AMPs and pneumonia protein as determined by I-TASSER (https://zhanggroup.org/I-TASSER/)^29^ and visualized using PyMOL version 1.3^30^. 3D Structure of (**a**) Respiratory syncytial virus X fusion core protein (**b1**) alpha-helical AMP, (**b2**) beta-sheet AMP, (**b3**) extended sheet AMP.

Representative figures from the I-TASSER server after predicting the 3-D structures of the anti-pneumonia AMPs (ligands) and the protein receptors are shown in Fig. 1. The results demonstrate that all AMPs predicted showed different secondary structures, including α-helices, parallel β-sheet, anti-parallel β-sheet, extended, and loop conformational structures.

For structure prediction assessment utilizing I-TASSER (Table 7), a few parameters, for example, Confidence score (C-score), Template modeling score (TM-score), and Root Mean Square Deviation (RMSD), were utilized for the prediction of the putative AMPs and pneumonia protein receptor 3-D structures. The results demonstrated that the C-score of all the anticipated 3-D structures for the anti-viral pneumonia AMPs and the pneumonia receptor proteins were between the estimations of − 5 to 2 (see Table7), which suggests an existing template by I TASSER for their structure prediction^40^. The determined C-score of BOPAM-RSV11 was lower than that of the other AMPs and could show that this molecule had no accessible template for prediction by I-TASSER but was not a random prediction^29^. TM-score has of late been proposed for estimating the structural compatibility between two structures^41^. A TM-score > 0.5 shows a model of right topology, and a TM-score < 0.17 implies irregular compatibility. From the results, the TM-score of the predicted structures of the AMPs and protein receptors was higher than the cut-off value of 0.5. This signifies that these structures had a correct topology with structural similarity to the templates that were used to predict their structures^29,41^. Although there is no defined RMSD value for 3-D structure prediction, an RMSD value of 2–4 Å is considered good, and an RMSD ≤ 1 Å is considered ideal. Thus, all anti-viral pneumonia AMPs and the receptor proteins having RMSD within the accepted range (Table 7) had less distance and the atomic deviation between the peptides and the templates used for their 3-D structure prediction^42,43^.

**Table 7:** Quality assessment scores of the predicted 3-D structures of the pneumonia receptors and the putative anti-pneumonia AMPs.

S/N	Anti-Pneumonia proteins and the Putative AMPs	C-score	TM Score	RSMD
1	INFA Nucleoprotein	0.98	0.85 ± 0.08	3.2 ± 1.3 Å
2	INFB Nucleoprotein	− 0.96	0.59 ± 0.14	3.8 ± 1.6 Å
3	RSV Chain A Matrix protein	1.99	0.99 ± 0.04	2.0 ± 1.6 Å
4	BOPAM-INFA1	− 1.18	0.57 ± 0.15	2.5 ± 1.9 Å
5	BOPAM-INFA2	− 0.21	0.69 ± 0.12	0.8 ± 0.8 Å
6	BOPAM-INFA3	− 1.61	0.57 ± 0.15	2.4 ± 1.8 Å
7	BOPAM-INFA4	− 1.40	0.54 ± 0.15	2.9 ± 2.1 Å
8	BOPAM-INFA5	− 1.21	0.56 ± 0.15	2.5 ± 1.9 Å
9	BOPAM-INFA6	− 1.19	0.57 ± 0.15	2.5 ± 1.9 Å
10	BOPAM-INFA7	− 1.11	0.58 ± 0.14	2.3 ± 1.8 Å
11	BOPAM-INFA8	− 1.14	0.57 ± 0.15	2.4 ± 1.8 Å
12	BOPAM-INFB1	− 0.56	0.64 ± 0.13	1.5 ± 1.4 Å
13	BOPAM-INFB2	− 0.57	0.64 ± 0.13	1.5 ± 1.4 Å
14	BOPAM-INFB3	− 1.09	0.58 ± 0.14	2.4 ± 1.8 Å
15	BOPAM-INFB4	0.16	0.73 ± 0.11	0.5 ± 0.5 Å
16	BOPAM-INFB5	− 0.33	0.67 ± 0.13	1.1 ± 1.1 Å
17	BOPAM-INFB6	− 1.12	0.57 ± 0.14	2.5 ± 1.9 Å
18	BOPAM-RSV1	− 0.03	0.71 ± 0.12	0.7 ± 0.7 Å
19	BOPAM-RSV2	0.02	0.72 ± 0.11	0.6 ± 0.6 Å
20	BOPAM-RSV3	− 0.03	0.71 ± 0.12	0.7 ± 0.7 Å
21	BOPAM-RSV4	− 1.20	0.56 ± 0.15	2.8 ± 2.0 Å
22	BOPAM-RSV5	− 0.62	0.63 ± 0.13	1.7 ± 1.5 Å
23	BOPAM-RSV6	− 0.18	0.69 ± 0.12	1.0 ± 1.0 Å
24	BOPAM-RSV7	− 0.10	0.70 ± 0.12	0.8 ± 0.8 Å
25	BOPAM-RSV8	− 1.24	0.56 ± 0.15	2.8 ± 2.1 Å
26	BOPAM-RSV9	− 0.74	0.62 ± 0.14	1.9 ± 1.6 Å
27	BOPAM-RSV10	− 1.10	0.58 ± 0.14	2.6 ± 1.9 Å
28	BOPAM-RSV11	− 1.63	0.52 ± 0.15	3.6 ± 2.5 Å
29	BOPAM-RSV12	− 1.42	0.54 ± 0.15	3.2 ± 2.2 Å
30	BOPAM-RSV13	− 0.14	0.70 ± 0.12	0.9 ± 0.9 Å

*S/N* serial number, *INFA* Influenza A virus, *INFB* Influenza B virus; *RSV* Respiratory Syncytial virus. *BOPAM-INFA* anti-Influenza A virus AMPs, *BOPAM-INFB* Anti-Influenza B virus AMPs, *BOPAM-RSV* anti-Respiratory Syncytial virus AMPs, *C-score* Confidence score; *TM score* Template Modeling score, *RMSD* root-mean-square deviation.

RMSD is sensitive to local error since it is an average distance of all residue sets in two structures, hence the for proposing TM-score. For example, a misorientation of the structure will increase the RMSD value even though the global topology of the structure is right. TM-score is not sensitive to misorientation in the region of the residues, which makes the score insensitive toward the local modelling mistake and, in this manner, a more reliable measure.

### Docking interaction analysis of the putative anti-pneumonia amps with viral pneumonia receptors

**Figure 2:**
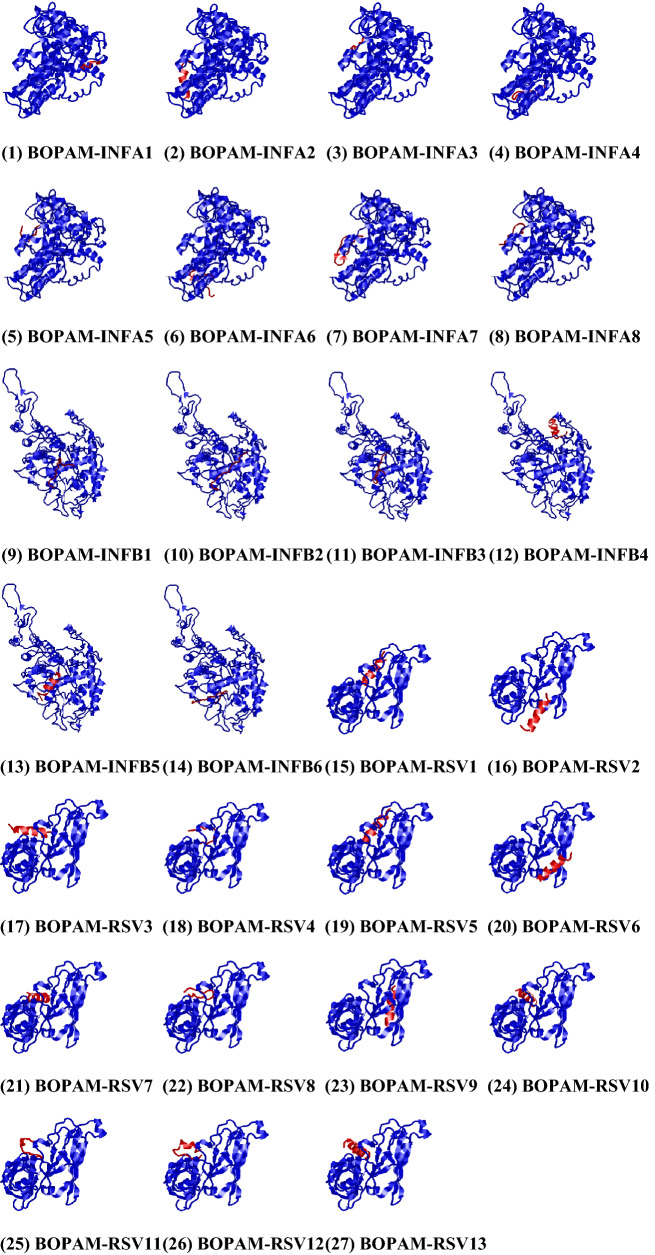
Docking interaction of the pneumonia protein receptors and putative anti-pneumonia AMPs produced from PATCHDOCK (https://bioinfo3d.cs.tau.ac.il/PatchDock/php.php)^32^ and visualized using PyMOL version 1.3^30^.. The structure in colour blue represents the receptor proteins with the ligand depicted in red with their orientations in their respective receptor.

The output figures from the PATCHDOCK and HDock servers after predicting the docking interaction between the anti-pneumonia AMPs (ligands) and the protein receptors were analyzed (Fig. 2). The spatial docking interaction analysis indicated that all the AMPs bound firmly to their proteins. Also, the computational investigation was done to affirm the AMPs with the most binding potential. These amino acid residues partook in the complex formation and towards which terminal of the proteins the binding occurs. Among the anti-Influenza A AMPs, only BOPAM-INFA1 bound at a different orientation to the nucleoprotein receptor. In contrast, BOPAM-INFB4 bound differently to the influenza B nucleoprotein when compared to other anti-Influenza B AMPs. All anti-Respiratory syncytial virus AMPs are bound on the same chain A fusion protein orientation except BOPAM-RSV2, 6, and 9.

BOPAM-RSVs bound more firmly to chain A protein with the highest binding geometry score noticed for BOPAM-RSV4. In a similar vein, the BOPAM-INFAs bound more firmly to nucleoprotein with the most binding geometry score noticed for BOPAM-INFA4. Also, in Table 8, BOPAM-INFA5, BOPAM-INFB4, and BOPAM-RSV4 have the highest area scores of 1601.80, 1740.90, and 1244.20, respectively, which denote the approximate interface area of their complexes to their respective receptors. It is also observed that BOPAM-INFA7, BOPAM-INFB6, and BOPAM-RSV8 have the lowest ACE scores of − 474.03, − 259.94, and − 368.59, which is the desolvation free energy needed for the ligand to shift atoms from water to the interior of the protein receptors^44^.

**Table 8:** Quality assessment scores of the docking analysis for the anti-pneumonia putative AMPs and the pneumonia receptors.

S/N	Receptors	Ligands	Binding scores	Area	ACE
1	Nucleoprotein	BOPAM-INFA1	12,134	1401.00	− 180.40
2	Nucleoprotein	BOPAM-INFA2	10,870	1487.40	− 345.63
3	Nucleoprotein	BOPAM-INFA3	10,572	1511.30	− 348.97
4	Nucleoprotein	BOPAM-INFA4	12,300	1522.10	− 418.43
5	Nucleoprotein	BOPAM-INFA5	11,778	1601.80	− 463.71
6	Nucleoprotein	BOPAM-INFA6	12,110	1551.00	− 42.13
7	Nucleoprotein	BOPAM-INFA7	10,982	1528.10	− 474.03
8	Nucleoprotein	BOPAM-INFA8	12,604	1583.00	− 441.39
9	Nucleoprotein	BOPAM-INFB1	12,816	1669.40	− 90.16
10	Nucleoprotein	BOPAM-INFB2	13,042	1706.50	105.76
11	Nucleoprotein	BOPAM-INFB3	11,606	1594.60	1.44
12	Nucleoprotein	BOPAM-INFB4	12,028	1740.90	− 153.56
13	Nucleoprotein	BOPAM-INFB5	11,704	1637.10	− 15.45
14	Nucleoprotein	BOPAM-INFB6	11,584	1564.00	− 259.94
15	Chain A Fusion Protein	BOPAM-RSV1	8906	1135.20	− 39.10
16	Chain A Fusion Protein	BOPAM-RSV2	8702	1023.90	− 56.69
17	Chain A Fusion Protein	BOPAM-RSV3	9068	1162.30	94.22
18	Chain A Fusion Protein	BOPAM-RSV4	9522	1244.20	− 176.85
19	Chain A Fusion Protein	BOPAM-RSV5	8274	1021.20	237.76
20	Chain A Fusion Protein	BOPAM-RSV6	8194	1033.90	− 150.77
21	Chain A Fusion Protein	BOPAM-RSV7	8784	1017.30	154.74
22	Chain A Fusion Protein	BOPAM-RSV8	8820	1082.10	− 368.59
23	Chain A Fusion Protein	BOPAM-RSV9	8500	1061.90	106.65
24	Chain A Fusion Protein	BOPAM-RSV10	9020	1114.50	− 47.18
25	Chain A Fusion Protein	BOPAM-RSV11	8424	1082.80	8.20
26	Chain A Fusion Protein	BOPAM-RSV12	8602	1112.30	264.91
27	Chain A Fusion Protein	BOPAM-RSV13	8866	1079.40	187.72

*S/N* serial number, *ACE* atomic contact energy.

The putative anti-influenza A AMPs displayed a high docking energy score using HDock, with BOPAM-INFA8 showing the highest energy − 199 kJ/mol. Similarly, all anti-influenza B AMPs displayed high binding energy to their receptors, with BOPAM-INFB2 having the highest docking energy score. Anti-respiratory syncytial virus AMPs showed high energy docking energy scores, with BOPAM-RSV4 and 3 having the highest docking energy scores to the receptor protein. The root-mean-square values are also generated from the HDock server as indicated in Table 9 alongside the hotspot interacting residues of the anti-viral pneumonia AMPs and their respective receptor proteins. The result from the HDock server shows consistency when compared to the PatchDock server.

**Table 9:** Quality assessment scores of the docking analysis from HDock for the anti-pneumonia putative AMPs and the pneumonia receptors with the hotspot interacting residues.

AMPs	Docking score (Kj/mol)	RMSD	Interface residues in the receptor	Interface residues in the ligand
BOPAM-INFA1	− 185.97	83.37	E18 A22 I25 R26 V29 S297 L298 V299 G300 D302 I388 R389 T390 R391 S392 G394 N395 S457 F458 Q459 G460 R461 F464 A471	T1 P2 F4 I5 D6 G7 Q8 V9 P10 I11 P12 K13 Q14
BOPAM-INFA12	− 165.06	77.48	R74 Y78 E80 E81 R150 R152 A153 R156 T157 S170 R174 M191 E192 R195 M196 R195 D203 F101 N211 T216	C1 P2 I4 L5 D6 A8 I9 Q10 L12 P13 K14
BOPAM-INFA3	− 178.81	72.93	R65 Y78 E81 H82 P83 S84 A85 G86 K87 G93 P95 Y97 R106 L108 I109 L110 N144 Y148 Q149 R150 T151 R152 A153 T171 R317 Q364 I365 A366 S367 E369	T1 P2 T3 F4 I5 D6 G7 Q8 V9 T10 V11 P12 D13 Q14
BOPAM-INFA4	− 190.10	86.62	M1 T3 F71 D72 Q73 R74 R75 N76 E80 T130 R174 R175 S176 G177 A178 A181 E192 M196 R199 F206 N211 R214 T215 A218 M222	T1 P2 T3 F4 I5 D6 G7 Q8 V9 P10 M11 P12 Q13 Q14
BOPAM-INFA5	− 188.34	69.94	I57 Q58 L61 T62 R65 Y78 E81 H82 P95 Y97 N144 Y148 R150 R152 A153 R156 S170 T171 M191 R195 V363 Q364 I365 A366 S367 N368	T1 F4 I5 D6 G7 Q8 V9 P10 I11 P12 Q13 Q14
BOPAM-INFA6	− 179.78	87.02	M1 A2 T3 Y10 E73 R74 E80 R174 R175 S176 G177 A178 A181 M196 N211 R213 R214 T215 I217 A218 R221 M222 I225	T1 P2 K3 F4 I5 D6 G7 Q8 V9 P10 I11 P12 E13
BOPAM-INFA7	− 190.23	69.59	K77 Y78 E81 H140 S141 N144 Y148 R152 A153 R156 T157 S170 T171 L172 P173 R174 R195 K198 R199 N202 D203	C1 P2 I4 D6 S7 S8 I9 Q10 V11 F12 P13 K14
BOPAM-INFA8	− 199.39	71.50	T62 R65 Y78 E81 H82 P83 P95 Y97 Q144 Y148 R150 R152 A153 R156 T171 R317 G362 Q364 I365 A366 S367 N368	T1 P2 T3 F4 I5 D6 G7 Q8 V9 I11 P12 E13 Q14
BOPAM-INFB1	− 231.34	52.74	R116 F129 K132 N134 D137 H201 N205 F209 R211 T232 L233 P234 R235 E253 R256 F257 R260 L267 R269 D270 K272 A273 A276 Y277 I280 H420 P422	D2 S4 H5 R6 W7 T8 F9 L10 R11 V12 P13 P14 Q15
BOPAM-INFB2	− 244.92	50.92	S2 T11 H112 R116 D123 F129 K132 N134 R136 D137 E140 M198 H201 M204 N205 C208 F209 R211 S231 T232 L233 P234 R235 R236 G238 R256 K272 R373 G418 F419 H420	M1 D2 V3 S4 H5 R6 W7 T8 F9 L10 M11 P13
BOPAM-INFB3	− 218.75	55.13	F129 K132 K133 N134 R136 D137 E140 R211 A214 S231 T232 L233 P234 R235 G238 A239 V242 K245 L250 E253 R256 F257 R260 L267 K272 A273 T275 A276 Y277 K279 I280 N283	L1 N2 C3 N4 P5 Q6 L7 L8 C9 L10 N11 C12 N13 P14 Q15
BOPAM-INFB4	− 205.31	65.69	R34 P35 L38 V62 G63 R64 T66 Q67 K69 Q70 T71 P72 T73 E74 I75 K76 K331 S353 V355 G356 P444 M445 T446 R447 F517 G519 K520 K521 F523 N529 K530 T531 P533	L1 K2 L4 Q5 L6 L8 F9 L10 K11 V12 P13 Q14 L15
BOPAM-INFB5	− 222.41	58.11	F129 K132 K133 N134 A135 R136 D137 V138 E140 R211 K213 A214 L215 R217 V218 F228 S231 T232 L233 P234 R235 K245 A252 E253 R256 F257 R260 L267 A273 A276 I280	L1 T2 I3 L4 Q5 L6 L7 L8 F9 L10 K11 V12 P13 Q14 L15
BOPAM-INFB6	− 211.90	64.35	H112 R116 L119 A120 D123 K125 F129 N134 R136 D137 E140 M198 H201 S202 M204 N205 C208 F209 Q210 R211 K213 S231 T232 L233 P234 R411 S412 H420 F547	L1 N2 C3 N4 P5 P6 L7 L8 C9 L10 N11 C12 N13 Q15
BOPAM-RSV1	− 179.68	42.54	W37 F90 T91 I92 C93 V96 S97 V131 K132 D133 T135 M136 T138 L139 N140 P141 Y165 S168 Y199 S200 L203 D227 G229 A230 Y231	I1 V2 S3 S4 I5 K6 E7 I9 N10 C12 K13 F16
BOPAM-RSV2	− 181.14	50.32	E1 F2 M3 E4 T5 Y6 V7 N8 A116 I154 K195 I196 I197 P198 L202 V240 T241 T242 N243 W244 K245 H246	E1 V2 I5 K8 I9 S11 N12 L13 S14 T15 V16
BOPAM-RSV3	− 201.34	30.38	V38 P39 M40 F41 P87 K89 F90 T91 K125 L128 T130 V131 K132 D133 T164 Y165 Y199 S200 L203 I225 V226 D227 A230 Y231	I2 V3 N6 K7 I9 D10 N12 T13 T14 A15 I16
BOPAM-RSV4	− 201.29	42.59	W37 V38 P39 M40 K68 F90 T91 I92 C93 N95 V96 V131 K132 T135 Y165 Y199 S200 G201 D227 G229 A230 Y231 E233	K1 T3 E4 N6 N8 I9 D10 N11 N12 V13 N14 I15 I16
BOPAM-RSV5	− 179.82	41.22	W37 F90 I92 C93 V96 T130 V131 K132 D133 T135 M136 T138 L139 P141 Y165 S168 Y199 S200 L203 D227 L228 G229 A230 Y231 M256	A1 I2 N3 S4 V5 S6 V9 N10 N12 T13 I16
BOPAM-RSV6	− 163.11	45.62	E4 T5 Y6 V7 N8 L10 N23 A116 C117 I154 T192 N193 A194 K195 I196 P198 Y238 T241 T242 N243 K245 H246 T247 A248 T249	K1 F2 Q4 I5 C6 A8 I9 Q12 T13 I16
BOPAM-RSV7	− 178.67	39.42	W37 V38 P39 M40 K68 G69 F90 T91 I92 C93 N95 V96 K125 L128 V131 K132 Y199 S200 I225 D227 G229 A230 E233	N1 I2 S3 N4 V5 K6 N7 E8 L9 N10 Q11 I13 N14 N15
BOPAM-RSV8	− 192.49	35.34	W37 P39 M40 F41 Q42 K68 F90 T91 I92 C93 V96 K125 L128 T130 V131 K132 D133 Y199 S200 L203 I225 V226 D227 G229 A230 Y231	S1 I2 N4 F5 N6 E7 N8 S9 N10 V11 L12 L13 S14 A15 V16
BOPAM-RSV9	− 175.75	53.01	D144 I146 N153 T156 K158 K159 V160 I161 I162 P163 Y165 A230 Y231 L232 E233 K234 E235 W244 K254 M256 E257	N1 I2 K4 V5 T6 Q8 V9 N12 T13 I16
BOPAM-RSV10	− 174.39	38.74	W37 P39 M40 F41 K68 G69 P70 P87 F90 T91 C93 N95 V96 K125 L128 T130 V131 K132 D133 Y199 S200 I225 D227 A230 Y231 E233	I2 N3 D4 V5 S6 K7 V9 N10 T11 N12 T13 T14 N15 I16
BOPAM-RSV11	− 163.37	35.24	W37 V38 P39 M40 F41 K68 F90 T91 I92 C93 V96 K125 T130 K132 Y199 S200 L203 I225 V226 D227 G229 A230	N1 V2 K8 V9 D10 V11 N12 T13 A14 D15 I16
BOPAM-RSV12	− 174.81	33.98	W37 P39 M40 F41 T66 K68 S71 R73 P87 K89 F90 T91 I92 C93 V96 K125 L128 T130 K132 D133 Y199 S200 I225 D227 G229 A230	D1 I2 T3 N4 N6 N7 I9 A11 K12 F13 T14 K15 I16
BOPAM-RSV13	− 186.37	31.78	W37 P39 M40 F41 Q42 K68 P87 F90 T91 C93 N95 V96 K125 N126 L128 T130 V131 K132 D133 Y199 S200 L203 I225 D227 A230 Y231	K1 I2 D4 L5 N6 K8 L9 D10 R12 I13 N15 V16

## Discussion

Experimentally validated AMPs were utilized for model construction in this research because their activities have been established since they had demonstrated activity against the target pneumonia viruses with the minimum inhibitory concentration (MIC) as an indicator using the agar dilution or broth micro-dilution strategies, as indicated in the databases^45^. The list of anti-pneumonia AMPs was retained in their separate pathogenic target groups as recovered from the different databases to take into account specific species/microbe profile creation. Also, the profile creation step using the training dataset was carried out to train the HMMER software to assess the discriminatory capacity and quality of the AMPs profiles with both positive (test) and negative (neuropeptides) datasets. This technique of utilizing random sequences as positive and negative datasets is a regularly used method. It depends on the presumption that the probability of discovering random sequences with a discriminative propensity is exceptionally low^29^. Assessment of the profiles’ performance showed that they were specificity, accuracy, sensitivity, with excellent MCC^43^. The relatively low sensitivity of INFA suggests there was an overlap in the conserved domains of its AMPs [45. This outcome is in line with the work of Bhadra, Yan (48), where performance was compared using sensitivity, specificity, accuracy, and MCC employing benchmark datasets as inputs. Scanning the profiles to recognize novel anti-viral AMPs, profile INFA yielded eight anti-Influenza A AMPs profile INFB yielded six anti-Influenza B AMPs while RSVM yielded 13 anti-Respiratory Syncytial virus AMPs (Table 4). The HMMER reported credible E-values for the AMPs to capture the sequences’ diversity since the input AMPs were derived from various life forms^46^. There was an exceptionally low probability that these peptides were wrongly predicted to be anti-pneumonia AMPs.

Besides, some protein chains, for example, fusion protein core A, which are integral RNA proteins of Respiratory syncytial virus, mediate passage into the transmembrane glycoproteins of the host cell to elicit apoptosis^38^. They additionally assume a pivotal function in the virus assembly and interact with the RNA complex and the viral membrane. Recognition of these proteins in the body fluid has indicated just slight antigenic variance, which is not progressive, a significant factor for their utilization in detecting the virus^47^. Influenza A and B nucleoproteins play some significant structural and functional roles that could be investigated for their diagnostics. They are bi-functional membrane/RNA-binding proteins that participate in the encapsulation of the RNA-nucleoprotein core of the membrane envelope [56]. These nucleoproteins have been utilized in the diagnosis of pneumonia [56]. The utilization of receptor protein applicants, for example, Respiratory syncytial virus fusion protein chains A^48^, *Influenza* A virus nucleoprotein^49,50^, and *Influenza* B virus nucleoprotein^51^ in the diagnosis of pneumonia is justified because they are synthesized in generally high concentration inside body fluid across all strains and subtypes of these microorganisms; do not change with time; abundantly available either as cell surface receptor and moderately stable to a gentle in vitro handling.

Moreover, the presence of charged, polar, and non-polar amino acids in the putative anti-viral AMPs and the viral receptors is the conferment of charge, improved hydrophobicity, and increased binding potential on them. The hydrophobicity result of the AMPs lower than 30% is not an ideal physicochemical parameter because decreased hydrophobicity results in poor peptide helicity, diminished self-associating capacity in aqueous conditions, and poor antimicrobial activity^52^. Decreased hydrophobicity observed for BOPAM-INFA1, 3, 4, 6, and 8 is an outcome of polar amino acids, giving them antimicrobial activities. As of late, AMPs from sugar‐functionalized phosphonium polymers have been reported to require the hydrophilic part of their molecular structure to exert antibacterial activities against Gram‐negative *Escherichia coli* Gram‐positive *Staphylococcus aureus*^53^. All the AMPs had significant physicochemical parameters that made them bona vide AMPs in charge and the Boman index. The utilization of physicochemical parameters as indices to assess AMPs is in concurrence with the work by Hollmann, Martinez (53) where a re-assessment of the physicochemical properties of antimicrobial peptides was evaluated, bringing about a characteristic thermal change profile in model vesicles which was utilized to rank novel molecules with unknown biological action.

The structure prediction results generated for the AMPs and the receptors are in accordance with the different structural conformations displayed by known AMPs and proteins. Examples of known AMPs and their structures are tachyplesin from horseshoe crabs and bovine lactoferricin, which have beta-sheet conformations; magainin simple and melittin having alpha-helical conformations. The C-score from I-TASSER is a measure of the certainty of the modeling template used for the prediction to anticipate the quality of the structure, that is, the distance between the anticipated model and the local structures^41^. Both TM and RMSD scores are known standards for estimating structural closeness between two structures for accuracy of structural model when the local structure is known^30^. The peptides’ structures were predicted, and the outcomes demonstrated that these peptides conformed to known AMPs. In any case, the AMPs are thought to be putative anti-pneumonia peptides because of the absence of wet laboratory experiments for these molecules. This outcome relates to the work of Tincho, Gabere (12), where binding geometry scoring was utilized as the criteria in the determination of applicant AMPs for HIV diagnostics. These perceptions were additionally affirmed utilizing an in-house lateral flow device in which the putative AMPs were utilized to recognize HIV in patient samples^13^.

The anti-viral AMPs also displayed high binding energy scores with the viral pneumonia receptors using PatchDock and HDock servers. Both servers use scoring functions to simulate ligands’ conformations on protein receptors. HDock server utilizes the classical force-fields-based scoring function to estimate and assess the non-bonded interactions (electrostatic and van der Waals). The docking interaction analysis of the AMPs revealed that all AMPs bound the respective viral receptors with a high binding capacity with BOPAM-INFA8, BOPAM-INFB2, and BOPAM-RSV4 having the highest binding potential and area with the most reduced atomic contact energy (Tables 8 and 9). Comparing these results with the physicochemical results in Table 5, BOPAM-INFA1, 2, 7, BOPAM-INFB3, 6, BOPAM-RSV3, 7, 12, and 13, which indicated zero net charges, gave the most reduced binding affinities (Boman index values) with the pneumonia receptor proteins. The result from this research showed BOPAM-INFA8, BOPAM-INFB4, and BOPAM-RSV4 as the best applicant specialists for the detection of the respective viral pneumonia pathogens. This binding affinity and other parameters, for example, area and atomic contact energy^2^, are significant in determining novel anti-viral AMPs for potential use in pneumonia diagnosis through the development of an LFD.

Designing and modeling novel AMPs for diagnostics is an active area of research to reduce the abuse of the conventional antibiotic agents and mitigate the non-specificity of the current diagnostic and prognostic biomarkers. One limitation for HMMER’s use is the data correlation with the amino acid residues of AMPs which is hard to capture by this software because of the linear nature of HMM profile. An example of such data correlation is predicting the actual distance between the folding of proteins, their spreading out; and the forecast between the electrical and chemical connectivity. Another constraint is the low sensitivity of HMMER to the utilization of small datasets due to the accessible number of AMPs in the databases to specific targets. Also, AMPs are not advisable for use when proteolytic degradation is possible due to L-amino acids’ presence in them^54^. All these limitations were taken into consideration during the design of this work to ensure that the sensitive detection of the viral pneumonia utilizing anti-viral AMPs was not compromised. The use of the putative AMPs from this analysis would greatly benefit the diagnosis of viral pneumonia through the HMMER’s utilization in the prediction of AMPs for model predictions. One of this work’s qualities is that it would offer knowledge into the modular architecture of AMPs utilizing in silico technologies for potential pneumonia diagnosis. This attempt offers promising perspectives for patients living with these conditions to develop accommodating lifestyles through sensitive detection of the viral pneumonia pathogens and would allow medical practitioners towards correct treatment plans.

## Conclusion

This research work distinguished novel AMPs for the potential detection of viral pneumonia utilizing the HMMER in silico technology, where 27 anti-viral peptides were generated. The putative anti-pneumonia AMPs demonstrated conformity to other known AMPs regarding their physicochemical qualities estimated by APD3 and BACTIBASE. This demonstrative framework’s fundamental goal is to facilitate the quest for specific biomarkers for the early recognition of viral pneumonia. Thus, the AMPs have indicated an incredible potential in evading the current diagnostic frameworks’ downsides. This research could be sought after molecular validation through the binding test of these AMPs with the viral proteins individually, utilizing an “on/off” binding test in an LFD setting to build up a model with these AMPs.

## Future work

Future work will incorporate the site-directed mutagenesis of the putative AMPs to upgrade them into more potent competitor diagnostic molecules. This analysis would be followed by an in vitro investigation of the anti-pneumonia activity of the transformed peptides. Furthermore, the EC50 of the AMPs and their selective index will be evaluated for the streamlined AMPs. The anti-pneumonia potential of these AMPs will be done on various pseudotypes of the pneumonia microbes to decide their diagnostic potential. Finally, the complex formed between the microbe receptors and putative AMPs will be unraveled utilizing structural biology to approve the perceptions made by the in silico binding examination.

## Supplementary Information

Supplementary Information.
