# Supplementary material for: Building HMM and molecular docking analysis for the sensitive detection of anti-viral pneumonia antimicrobial peptides (AMPs)
Source: Sci Rep. 2021 Oct 18;11:20621. doi: 10.1038/s41598-021-00223-8 (PMC8523717; doi:10.1038/s41598-021-00223-8)
Supplement: Supplementary file 1 — Supplementary Information. [file 41598_2021_223_MOESM1_ESM.zip › Supplemetary final/AMP INFLU A dataset.docx]

| ID | AMP | SOURCE | TARGET | SEQUENCE | REFERENCE |
| --- | --- | --- | --- | --- | --- |
| CAMPSQ350 | Alloferon-1 | Calliphora vicina [Blue blowfly] | Influenzae virus ( MIC = 25 microg ) | HGVSGHGQHGVHG=13 | [Proc Natl Acad Sci U S A.](https://www.ncbi.nlm.nih.gov/pubmed/?term=12235362) 2002 Oct 1;99(20):12628-32. Epub 2002 Sep 16.Antiviral and antitumor peptides from insects.[Chernysh S](https://www.ncbi.nlm.nih.gov/pubmed/?term=Chernysh%20S%5BAuthor%5D&cauthor=true&cauthor_uid=12235362)1, [Kim SI](https://www.ncbi.nlm.nih.gov/pubmed/?term=Kim%20SI%5BAuthor%5D&cauthor=true&cauthor_uid=12235362), [Bekker G](https://www.ncbi.nlm.nih.gov/pubmed/?term=Bekker%20G%5BAuthor%5D&cauthor=true&cauthor_uid=12235362), [Pleskach VA](https://www.ncbi.nlm.nih.gov/pubmed/?term=Pleskach%20VA%5BAuthor%5D&cauthor=true&cauthor_uid=12235362), [Filatova NA](https://www.ncbi.nlm.nih.gov/pubmed/?term=Filatova%20NA%5BAuthor%5D&cauthor=true&cauthor_uid=12235362), [Anikin VB](https://www.ncbi.nlm.nih.gov/pubmed/?term=Anikin%20VB%5BAuthor%5D&cauthor=true&cauthor_uid=12235362), [Platonov VG](https://www.ncbi.nlm.nih.gov/pubmed/?term=Platonov%20VG%5BAuthor%5D&cauthor=true&cauthor_uid=12235362), [Bulet P](https://www.ncbi.nlm.nih.gov/pubmed/?term=Bulet%20P%5BAuthor%5D&cauthor=true&cauthor_uid=12235362). |
| AP02571 | Cycloviolacin VY1 (cyclotides, plants; XXC; 3S=S; UCBB1b) | *Viola yedoensis* | influenza A virus H1N1(IC50 2.27 ug/ml) | CGESCVFIPCITTVLGCSCSIKVCYKNGSIP=31 | Liu MZ, Yang Y, Zhang SX, Tang L, Wang HM, Chen CJ, Shen ZF, Cheng KD, Kong JQ, Wang W.2014[A cyclotide against influenza A H1N1 virus from Viola yedoensis]2014 Jun;49(6):905-12. [PubMed](http://www.ncbi.nlm.nih.gov/pubmed/25212039) |
| AP02846 | Urumin (frog, amphibians, animals; s; UCSS1a) | *Hydrophylax bahuvistara*, India, Asia | Influenza A Viruses MIC=3.8 uM | IPLRGAFINGRWDSQCHRFSNGAIACA=27 | Holthausen DJ, Lee SH, Kumar VT, Bouvier NM, Krammer F, Ellebedy AH, Wrammert J, Lowen AC, George S, Pillai MR, Jacob J. 2017. An Amphibian Host Defense Peptide Is Virucidal for Human H1 Hemagglutinin-Bearing Influenza Viruses. Immunity. 2017 Apr 18;46(4):587-595. [PubMed](http://www.ncbi.nlm.nih.gov/pubmed/28423338) |
| AVP0554 | EB | FGF-4 signal sequence, Orthomyxoviridae | INFV A  8μM[Virus entry](http://crdd.osdd.net/servers/avpdb/browse.php?by=Virus%20entry&TYPE=Target) | RRKKAAVALLPAVLLALLAP=20 | Jones JC, Turpin EA, Bultmann H, Brandt CR, Schultz-Cherry S. Inhibition of influenza virus infection by a novel antiviral peptide that targets viral attachment to cells. [J Virol.](https://www.ncbi.nlm.nih.gov/pubmed/17005658) 2006 Dec;80(24):11960-7. |
| AVP0961 | 18-c01 | Phage display | INFV A3.2μM | GWWYKGRARPVSAVA=15 | Matsubara T, Sumi M, Kubota H, Taki T, Okahata Y, Sato T.Inhibition of influenza virus infections by sialylgalactose-binding peptides selected from a phage library.[J Med Chem.](https://www.ncbi.nlm.nih.gov/pubmed/19558186) 2009 Jul 23;52(14):4247-56. doi: 10.1021 |
| AVP0962 | C18-c03 | Phage display | INFV A6.5μM | RAVWRHSVATPSHSV=15 | “ |
| AVP0963 | C18-c01W2A | Phage display | INFV A53μM | GAWYKGRARPVSAVA=15 | “ |
| AVP0964 | C18-c01P10A | Phage display | INFV A89μM | GWWYKGRARAVSAVA=15 | “ |
| AVP0965 | C18-c01r | “ | INFV A44μM | AVASVPRARGKYWWG=15 | “ |
| AVP0966 | C18-p1b | “ | INFV A52μM | DFRRLPGAFWQLRQP=15 | “ |
| AVP0967 | C18-cp3c | “ | INFV A66μM | AETVESCLAKPHTEN=15 | “ |
| AVP0968 | c01 | “ | INFV A  >500μM | GWWYKGRARPVSAVA=15 | “ |
| AVP0969 | c03 | “ | INFV A  >500μM | RAVWRHSVATPSHSV=15 | “ |
| AVP0977 | 45658 | INFV A polymerase (PB1) | INFV A1.8nM Replication | MDVNPTLLFLKVPAQNAISTTFPYT=19 | Wunderlich K, Mayer D, Ranadheera C, Holler AS, Manz B, Martin A, Chase G, Tegge W, Frank R, Kessler U, Schwemmle M. Identification of a PA-binding peptide with inhibitory activity against influenza A and B virus replication. [PLoS One.](https://www.ncbi.nlm.nih.gov/pubmed/19841738) 2009 Oct 20;4(10):e7517. |
| AVP0978 | 45717 | INFV A polymerase (PB1) | INFV A661.77nM | VNPTLLFLKVPAQNAISTTFPYT=23 | “ |
| AVP0979 | 45778 | INFV A polymerase (PB1) | INFV A483nM | PTLLFLKVPAQNAISTTFPYT=21 | “ |
| AVP0980 | 45839 | “ | INFV A>3000nM | LLFLKVPAQNAISTTFPYT=19 | “ |
| AVP0981 | 45901 | “ | INFV A>3000nM | FLKVPAQNAISTTFPYT=17 | “ |
| AVP0982 | 45962 | “ | INFV A>3000nM | KVPAQNAISTTFPYT=15 | “ |
| AVP0983 | 43831 | “ | INFV A33.8nM | MDVNPTLLFLKVPAQNAIST=20 | “ |
| AVP0984 | 43101 | “ | INFV A29.45nM | MDVNPTLLFLKVPAQNAI=18 | “ |
| AVP0985 | 42370 | “ | INFV A45.86nM | MDVNPTLLFLKVPAQN=16 | “ |
| AVP0986 | 42005 | “ | INFV A43.32nM | MDVNPTLLFLKVPAQ=15 | “ |
| AVP0987 | 41640 | “ | INFV A34.53nM | MDVNPTLLFLKVPA=15 | “ |
| AVP0988 | 41275 | “ | INFV A138.17nM | MDVNPTLLFLKVP=13 | “ |
| AVP0989 | 41609 | “ | INFV A644nM | MDVNPTLLFLKV=12 | “ |
| AVP0990 | 41579 | “ | INFV A899nM | MDVNPTLLFLK=11 | “ |
| AVP0991 | 41548 | “ | INFV A>3000nM | MDVNPTLLFL=10 | “ |
| AVP0992 | 41518 | “ | “ | MDVNPTLLF=9 | “ |
| AVP0993 | 41487 | “ | “ | MDVNPTLL=8 | “ |
| AVP0994 | 41456 | “ | “ | MDVNPTL=7 | “ |
| AVP0995 | 41426 | “ | “ | MDVNPT=6 | “ |
| AVP0996 | PB1-1-15 B | “ | “ | MNINPYPLFIDVPIQ=15 | “ |
| AVP0997 | PB1-1-15 A D2N,V3I,L14I | “ | INFV A6.69nM | MNINPTLLFLKVPIQ=15 | “ |
| AVP0998 | PB1-1-15 A L10I,K11D | “ | INFV A>3000nM | MDVNPTLLFIDVPAQ=15 | “ |
| AVP0999 | PB1-1-15 A D2N,V3I | “ | INFV A12.96nM | MNINPTLLFLKVPAQ=15 | “ |
| AVP1000 | PB1-1-15 A T6Y,L7F | “ | INFV A7.51nM | MDVNPYFLFLKVPAQ=15 | “ |
| AVP1001 | PB1-1-15 A L7F | “ | INFV A>3000nM | MDVNPTFLFLKVPAQ=15 | “ |
| AVP1002 | PB1-1-15 A T6Y | “ | INFV A21.64nM | MDVNPYLLFLKVPAQ=15 | “ |
| AVP1003 | PB1-1-15 A T6F | “ | INFV A2.84nM | MDVNPFLLFLKVPAQ=15 | “ |
| AVP1004 | PB1-1-15 A T6W | “ | INFV A3.4nM | MDVNPWLLFLKVPAQ=15 | “ |
| AVP1005 | PB1-1-15 A T6H | “ | INFV A292.16nM | MDVNPHLLFLKVPAQ=15 | “ |
| AVP1006 | PB1-1-15 A T6C | “ | INFV A43.58nM | MDVNPCLLFLKVPAQ=15 | “ |
| AVP1753 | FP3 | Mimetic for the SOCS protein | INFV A0.00003μM[Virus entry](http://crdd.osdd.net/servers/avpdb/browse.php?by=Virus%20entry&TYPE=Target) | WLVFFVIFYFFRRRKK=16 | Nicol MQ, Ligertwood Y, Bacon MN, Dutia BM, Nash AA. A novel family of peptides with potent activity against influenza A viruses.[J Gen Virol.](https://www.ncbi.nlm.nih.gov/pubmed/22258859) 2012 May;93(Pt 5):980-6. |
| AVP1754 | FP4 | “ | INFV A0.00004μM | RRKKWLVFFVIFYFFR=16 | “ |
| AVP1755 | FP2 | “ | INFV A0.00087μM | WLVFFVIAYFAR=12 | “ |
| AVP1756 | FP1 (Tkip) | “ | INFV A0.0936μM | WLVFFVIFYFFR=12 | “ |
| AVP1759 | FP7 | “ | INFV A0.15483μM | RRKKIFYFFR=10 | “ |
| AVP1806 | FP8 | “ | INFV A0.63806μM | WLVFFVRRKK=10 | “ |
| AVP1808 | FP9 | “ | INFV A1.48175μM | FFVIFYRRKK=10 | “ |
| AVP2028 | Peptide 6 | INFV A matrix protein 1 | INFV A0.7nM | CATCEQIADSQHRSHRQMV=19 | Judd AK, Sanchez A, Bucher DJ, Huffman JH, Bailey K, Sidwell RW. In vivo anti-influenza virus activity of a zinc finger peptide. [Antimicrob Agents Chemother.](https://www.ncbi.nlm.nih.gov/pubmed/9056014) 1997 Mar;41(3):687-92. |
| AVP2057 | Mucroporin-S2 | Scorpion venom | INFV A1.03μM | LFGLIPSLIGGLVSAFK=17 | Li Q, Zhao Z, Zhou D, Chen Y, Hong W, Cao L, Yang J, Zhang Y, Shi W, Cao Z, Wu Y, Yan H, Li W. Virucidal activity of a scorpion venom peptide variant mucroporin-M1 against measles, SARS-CoV and influenza H5N1 viruses. [Peptides.](https://www.ncbi.nlm.nih.gov/pubmed/21620914) 2011 Jul;32(7):1518-25. |
