# Supplementary material for: Building HMM and molecular docking analysis for the sensitive detection of anti-viral pneumonia antimicrobial peptides (AMPs)
Source: Sci Rep. 2021 Oct 18;11:20621. doi: 10.1038/s41598-021-00223-8 (PMC8523717; doi:10.1038/s41598-021-00223-8)
Supplement: Supplementary file 1 — Supplementary Information. [file 41598_2021_223_MOESM1_ESM.zip › Supplemetary final/AMP INFLU B dataset.docx]

| ID | AMP | SOURCE | TARGET | SEQUENCE | REFERENCE |
| --- | --- | --- | --- | --- | --- |
| [AVP1007](http://crdd.osdd.net/servers/avpdb/record.php?details=AVP1007) | PB1-1-15 A | INFV A polymerase (PB1) | INFV B IC50=>3000nM ([Replication](http://crdd.osdd.net/servers/avpdb/browse.php?by=Replication&TYPE=Target))  ELISA | MDVNPTLLFLKVPAQ=15 | [PLoS One.](https://www.ncbi.nlm.nih.gov/pubmed/19841738) 2009 Oct 20;4(10):e7517. doi: 10.1371/journal.pone.0007517.Identification of a PA-binding peptide with inhibitory activity against influenza A and B virus replication.[Wunderlich K](https://www.ncbi.nlm.nih.gov/pubmed/?term=Wunderlich%20K%5BAuthor%5D&cauthor=true&cauthor_uid=19841738)1, [Mayer D](https://www.ncbi.nlm.nih.gov/pubmed/?term=Mayer%20D%5BAuthor%5D&cauthor=true&cauthor_uid=19841738), [Ranadheera C](https://www.ncbi.nlm.nih.gov/pubmed/?term=Ranadheera%20C%5BAuthor%5D&cauthor=true&cauthor_uid=19841738), [Holler AS](https://www.ncbi.nlm.nih.gov/pubmed/?term=Holler%20AS%5BAuthor%5D&cauthor=true&cauthor_uid=19841738), [Mänz B](https://www.ncbi.nlm.nih.gov/pubmed/?term=Mänz%20B%5BAuthor%5D&cauthor=true&cauthor_uid=19841738), [Martin A](https://www.ncbi.nlm.nih.gov/pubmed/?term=Martin%20A%5BAuthor%5D&cauthor=true&cauthor_uid=19841738), [Chase G](https://www.ncbi.nlm.nih.gov/pubmed/?term=Chase%20G%5BAuthor%5D&cauthor=true&cauthor_uid=19841738), [Tegge W](https://www.ncbi.nlm.nih.gov/pubmed/?term=Tegge%20W%5BAuthor%5D&cauthor=true&cauthor_uid=19841738), [Frank R](https://www.ncbi.nlm.nih.gov/pubmed/?term=Frank%20R%5BAuthor%5D&cauthor=true&cauthor_uid=19841738), [Kessler U](https://www.ncbi.nlm.nih.gov/pubmed/?term=Kessler%20U%5BAuthor%5D&cauthor=true&cauthor_uid=19841738), [Schwemmle M](https://www.ncbi.nlm.nih.gov/pubmed/?term=Schwemmle%20M%5BAuthor%5D&cauthor=true&cauthor_uid=19841738). |
| AVP1008 | PB1-1-15 B | INFV A polymerase (PB1)**U** | Influenza B virus (INFV B)  45nM Replication | MNINPYPLFIDVPIQ=15 | Identification of a PA-binding peptide with inhibitory activity against influenza A and B virus replication.Wunderlich K, Mayer D, Ranadheera C, Holler AS, Manz B, Martin A, Chase G, Tegge W, Frank R, Kessler U, Schwemmle M.PLoS One. 2009 |
| AVP1009 | PB1-1-15 A D2N,V3I,L14I | INFV A polymerase (PB1) | INFV B>3000nM[Replication](http://crdd.osdd.net/servers/avpdb/browse.php?by=Replication&TYPE=Target) | **MNINPTLLFLKVPIQ=15** | [PLoS One.](https://www.ncbi.nlm.nih.gov/pubmed/19841738) 2009 Oct 20;4(10):e7517. doi: 10.1371/journal.pone.0007517.Identification of a PA-binding peptide with inhibitory activity against influenza A and B virus replication.[Wunderlich K](https://www.ncbi.nlm.nih.gov/pubmed/?term=Wunderlich%20K%5BAuthor%5D&cauthor=true&cauthor_uid=19841738)1, [Mayer D](https://www.ncbi.nlm.nih.gov/pubmed/?term=Mayer%20D%5BAuthor%5D&cauthor=true&cauthor_uid=19841738), [Ranadheera C](https://www.ncbi.nlm.nih.gov/pubmed/?term=Ranadheera%20C%5BAuthor%5D&cauthor=true&cauthor_uid=19841738), [Holler AS](https://www.ncbi.nlm.nih.gov/pubmed/?term=Holler%20AS%5BAuthor%5D&cauthor=true&cauthor_uid=19841738), [Mänz B](https://www.ncbi.nlm.nih.gov/pubmed/?term=Mänz%20B%5BAuthor%5D&cauthor=true&cauthor_uid=19841738), [Martin A](https://www.ncbi.nlm.nih.gov/pubmed/?term=Martin%20A%5BAuthor%5D&cauthor=true&cauthor_uid=19841738), [Chase G](https://www.ncbi.nlm.nih.gov/pubmed/?term=Chase%20G%5BAuthor%5D&cauthor=true&cauthor_uid=19841738), [Tegge W](https://www.ncbi.nlm.nih.gov/pubmed/?term=Tegge%20W%5BAuthor%5D&cauthor=true&cauthor_uid=19841738), [Frank R](https://www.ncbi.nlm.nih.gov/pubmed/?term=Frank%20R%5BAuthor%5D&cauthor=true&cauthor_uid=19841738), [Kessler U](https://www.ncbi.nlm.nih.gov/pubmed/?term=Kessler%20U%5BAuthor%5D&cauthor=true&cauthor_uid=19841738), [Schwemmle M](https://www.ncbi.nlm.nih.gov/pubmed/?term=Schwemmle%20M%5BAuthor%5D&cauthor=true&cauthor_uid=19841738). |
| [AVP1010](http://crdd.osdd.net/servers/avpdb/record.php?details=AVP1010) | PB1-1-15 A L10I,K11D | INFV A polymerase (PB1) | INFV B>3000nM[Replication](http://crdd.osdd.net/servers/avpdb/browse.php?by=Replication&TYPE=Target) | MDVNPTLLFIDVPAQ=15 | [PLoS One.](https://www.ncbi.nlm.nih.gov/pubmed/19841738) 2009 Oct 20;4(10):e7517. doi: 10.1371/journal.pone.0007517.Identification of a PA-binding peptide with inhibitory activity against influenza A and B virus replication.[Wunderlich K](https://www.ncbi.nlm.nih.gov/pubmed/?term=Wunderlich%20K%5BAuthor%5D&cauthor=true&cauthor_uid=19841738)1, [Mayer D](https://www.ncbi.nlm.nih.gov/pubmed/?term=Mayer%20D%5BAuthor%5D&cauthor=true&cauthor_uid=19841738), [Ranadheera C](https://www.ncbi.nlm.nih.gov/pubmed/?term=Ranadheera%20C%5BAuthor%5D&cauthor=true&cauthor_uid=19841738), [Holler AS](https://www.ncbi.nlm.nih.gov/pubmed/?term=Holler%20AS%5BAuthor%5D&cauthor=true&cauthor_uid=19841738), [Mänz B](https://www.ncbi.nlm.nih.gov/pubmed/?term=Mänz%20B%5BAuthor%5D&cauthor=true&cauthor_uid=19841738), [Martin A](https://www.ncbi.nlm.nih.gov/pubmed/?term=Martin%20A%5BAuthor%5D&cauthor=true&cauthor_uid=19841738), [Chase G](https://www.ncbi.nlm.nih.gov/pubmed/?term=Chase%20G%5BAuthor%5D&cauthor=true&cauthor_uid=19841738), [Tegge W](https://www.ncbi.nlm.nih.gov/pubmed/?term=Tegge%20W%5BAuthor%5D&cauthor=true&cauthor_uid=19841738), [Frank R](https://www.ncbi.nlm.nih.gov/pubmed/?term=Frank%20R%5BAuthor%5D&cauthor=true&cauthor_uid=19841738), [Kessler U](https://www.ncbi.nlm.nih.gov/pubmed/?term=Kessler%20U%5BAuthor%5D&cauthor=true&cauthor_uid=19841738), [Schwemmle M](https://www.ncbi.nlm.nih.gov/pubmed/?term=Schwemmle%20M%5BAuthor%5D&cauthor=true&cauthor_uid=19841738). |
| AVP1011 | PB1-1-15 A D2N,V3I | INFV A polymerase (PB1) | INFV B>3000nM[Replication](http://crdd.osdd.net/servers/avpdb/browse.php?by=Replication&TYPE=Target) | MNINPTLLFLKVPAQ=15 | Wunderlich K, Mayer D, Ranadheera C, Holler AS, Manz B, Martin A, Chase G, Tegge W, Frank R, Kessler U, Schwemmle M.Identification of a PA-binding peptide with inhibitory activity against influenza A and B virus replication.PLoS One. 20;4(10):e7517. Doi: 10.1371,2009 |
| AVP1012 | PB1-1-15 A T6Y,L7F | INFV A polymerase (PB1) | INFV B  345nM[Replication](http://crdd.osdd.net/servers/avpdb/browse.php?by=Replication&TYPE=Target) | MDVNPYFLFLKVPAQ=15 | “ |
| AVP1013 | PB1-1-15 A L7F | INFV A polymerase (PB1) | INFV B>3000nM[Replication](http://crdd.osdd.net/servers/avpdb/browse.php?by=Replication&TYPE=Target) | MDVNPTFLFLKVPAQ=15 | “ |
| [AVP1014](http://crdd.osdd.net/servers/avpdb/record.php?details=AVP1014) | PB1-1-15 A T6Y | INFV A polymerase (PB1) | INFV B>3000nM[Replication](http://crdd.osdd.net/servers/avpdb/browse.php?by=Replication&TYPE=Target) | MDVNPYLLFLKVPAQ=15 | “ |
| AVP1015 | PB1-1-15 A T6F | “ | INFV B  750nM[Replication](http://crdd.osdd.net/servers/avpdb/browse.php?by=Replication&TYPE=Target) | MDVNPFLLFLKVPAQ=15 | “ |
| AVP1016 | PB1-1-15 A T6W | “ | INFV B  628nM[Replication](http://crdd.osdd.net/servers/avpdb/browse.php?by=Replication&TYPE=Target) | MDVNPWLLFLKVPAQ=15 | “ |
| AVP1017 | PB1-1-15 A T6H | “ | INFV B  >3000nM[Replication](http://crdd.osdd.net/servers/avpdb/browse.php?by=Replication&TYPE=Target) | MDVNPHLLFLKVPAQ=15 | “ |
| AVP1018 | PB1-1-15 A T6C | “ | INFV B  >3000nM[Replication](http://crdd.osdd.net/servers/avpdb/browse.php?by=Replication&TYPE=Target) | MDVNPCLLFLKVPAQ=15 | “ |
