# Supplementary material for: Building HMM and molecular docking analysis for the sensitive detection of anti-viral pneumonia antimicrobial peptides (AMPs)
Source: Sci Rep. 2021 Oct 18;11:20621. doi: 10.1038/s41598-021-00223-8 (PMC8523717; doi:10.1038/s41598-021-00223-8)
Supplement: Supplementary file 1 — Supplementary Information. [file 41598_2021_223_MOESM1_ESM.zip › Supplemetary final/AMP RSV dataset.docx]

| ID | AMP | SOURCE | TARGET | SEQUENCE | REFERENCE |
| --- | --- | --- | --- | --- | --- |
| AP00281 | mCRAMP (mouse cathelin-related antimicrobial peptide; cathelicidin, animals; derivatives: CRAMP 18) | Mouse, *Mus musculus* | the single-stranded RNA virus respiratory syncytial virus (RSV) | GLLRKGGEKIGEKLKKIGQKIKNFFQKLVPQPEQ=34 | Identification of CRAMP, a cathelin-related antimicrobial peptide expressed in the embryonic and adult mouse.[Gallo RL](http://dermatology.ucsd.edu/faculty/Pages/gallo.aspx), Kim KJ, Bernfield M, Kozak CA, Zanetti M, Merluzzi L, Gennaro R. 1997, J. Biol. Chem. 1997: 272:13088-13093. [PubMed](http://www.ncbi.nlm.nih.gov/pubmed/9148921) |
| AP00310 | LL-37 [LL37; FALL-39; cathelicidin; UCLL1; human; chimpanzee; primates, mammals, animals; XXX; XXY; XXZ; BBBh2o, BBBm; BBMm, BBPP, BBN, BBL, BBR, JJsn; Derivatives: GF-17, IG-25, KR-12, etc. | neutrophils, moncytes; lymphocytes, Mesenchymal Stem Cells; islets; skin, sweat; airway surface liquid, saliva; *Homo sapiens*; Also *Pan troglodytes* | RSV | LLGDFFRKSKEKIGKEFKRIVQRIKDFLRNLVPRTES=37 | FALL-39, a putative human peptide antibiotic, is cysteine-free and expressed in bone marrow and testis.Agerberth B., Gunne H., Odeberg J., Kogner P., Boman HG., Gudmundsson GH.1995. Proc. Natl. Acad. Sci. U.S.A. 1995; 92:195-199 |
| AP02337 | RNase 2 (eosinophil-derived neurotoxin, EDN; EPX; Ribonuclease superfamily; UCSS1a; 4S=S, humans; primates, mammals, animals) | liver, lung, spleen, eosinophilic leukocytes; neutrophils, and monocytes, *Homo sapiens* | respiratory syncytial virus (RSV) | KPPQFTWAQWFETQHINMTSQQCTNAMQVINNYQRRCKNQNTFLLTTFANVVNVCGNPNMTCPSNKTRKNCHHSGSQVPLIHCNLTTPSPQNISNCRYAQTPANMFYIVACDNRDQRRDPPQYPVVPVHLDRII=131 | [Nucleic Acids Res.](https://www.ncbi.nlm.nih.gov/pubmed/9826755) 1998 Dec 1;26(23):5327-32.Evolution of antiviral activity in the ribonuclease A gene superfamily: evidence for a specific interaction between eosinophil-derived neurotoxin (EDN/RNase 2) and respiratory syncytial virus.[Domachowske JB](https://www.ncbi.nlm.nih.gov/pubmed/?term=Domachowske%20JB%5BAuthor%5D&cauthor=true&cauthor_uid=9826755)1, [Bonville CA](https://www.ncbi.nlm.nih.gov/pubmed/?term=Bonville%20CA%5BAuthor%5D&cauthor=true&cauthor_uid=9826755), [Dyer KD](https://www.ncbi.nlm.nih.gov/pubmed/?term=Dyer%20KD%5BAuthor%5D&cauthor=true&cauthor_uid=9826755), [Rosenberg HF](https://www.ncbi.nlm.nih.gov/pubmed/?term=Rosenberg%20HF%5BAuthor%5D&cauthor=true&cauthor_uid=9826755). |
| AP02338 | mEar2 (mouse eosinophil-associated ribonuclease 2, mRNase 2, Ribonuclease superfamily; mice; mammals, animals) | *Mus musculus* | single-stranded RNA virus respiratory syncytial virus (RSV) | LGQTPSQWFAIQHINNNANLQCNVEMQRINRFRRTCKGLNTFLHTSFANAVGVCGNPSGLCSDNISRNCHNSSSRVRITVCNITSRRRTPYTQCRYQPRRSLEYYTVACNPRTPQDSPMYPVVPVHLDGTF=132 | Moreau JM, Dyer KD, Bonville CA, Nitto T, Vasquez NL, Easton AJ, Domachowske JB, Rosenberg HF.2003,Diminished expression of an antiviral ribonuclease in response to pneumovirus infection in vivo.Antiviral Res. 2003 Aug;59(3):181-91.[PubMed](http://www.ncbi.nlm.nih.gov/pubmed/12927308); [Gene Bank](http://www.ncbi.nlm.nih.gov/protein/NP_031921.1); |
| AVP0127 | T-104 | RSV fusion (F) protein | RSV 91  EC50 (μM)[Fusion](http://crdd.osdd.net/servers/avpdb/browse.php?by=Fusion&TYPE=Target) | IINFYDPLVFPSDEFDASISQVNEKINQSLAFIRK=35 | [Proc Natl Acad Sci U S A.](https://www.ncbi.nlm.nih.gov/pubmed/8700906) 1996 Mar 5;93(5):2186-91.Peptides from conserved regions of paramyxovirus fusion (F) proteins are potent inhibitors of viral fusion.[Lambert DM](https://www.ncbi.nlm.nih.gov/pubmed/?term=Lambert%20DM%5BAuthor%5D&cauthor=true&cauthor_uid=8700906)1, [Barney S](https://www.ncbi.nlm.nih.gov/pubmed/?term=Barney%20S%5BAuthor%5D&cauthor=true&cauthor_uid=8700906), [Lambert AL](https://www.ncbi.nlm.nih.gov/pubmed/?term=Lambert%20AL%5BAuthor%5D&cauthor=true&cauthor_uid=8700906), [Guthrie K](https://www.ncbi.nlm.nih.gov/pubmed/?term=Guthrie%20K%5BAuthor%5D&cauthor=true&cauthor_uid=8700906), [Medinas R](https://www.ncbi.nlm.nih.gov/pubmed/?term=Medinas%20R%5BAuthor%5D&cauthor=true&cauthor_uid=8700906), [Davis DE](https://www.ncbi.nlm.nih.gov/pubmed/?term=Davis%20DE%5BAuthor%5D&cauthor=true&cauthor_uid=8700906), [Bucy T](https://www.ncbi.nlm.nih.gov/pubmed/?term=Bucy%20T%5BAuthor%5D&cauthor=true&cauthor_uid=8700906), [Erickson J](https://www.ncbi.nlm.nih.gov/pubmed/?term=Erickson%20J%5BAuthor%5D&cauthor=true&cauthor_uid=8700906), [Merutka G](https://www.ncbi.nlm.nih.gov/pubmed/?term=Merutka%20G%5BAuthor%5D&cauthor=true&cauthor_uid=8700906), [Petteway SR Jr](https://www.ncbi.nlm.nih.gov/pubmed/?term=Petteway%20SR%20Jr%5BAuthor%5D&cauthor=true&cauthor_uid=8700906). |
| AVP0128 | T-105 | *RSV fusion (F) protein* | RSV  93EC50 (μM)[Fusion](http://crdd.osdd.net/servers/avpdb/browse.php?by=Fusion&TYPE=Target) | INFYDPLVFPSDEFDASISQVNEKINQSLAFIRKS=35 | “ |
| AVP0129 | T-106 | *“* | RSV  >100EC50 (μM)[Fusion](http://crdd.osdd.net/servers/avpdb/browse.php?by=Fusion&TYPE=Target) | NFYDPLVFPSDEFDASISQVNEKINQSLAFIRKSD=35 | “ |
| AVP0130 | T-107 | *“* | RSV  20EC50 (μM) | FYDPLVFPSDEFDASISQVNEKINQSLAFIRKSDE=35 | “ |
| AVP0131 | T-108 | “ | RSV  6EC50 (μM)[Fusion](http://crdd.osdd.net/servers/avpdb/browse.php?by=Fusion&TYPE=Target) | YDPLVFPSDEFDASISQVNEKINQSLAFIRKSDEL=35 | “ |
| AVP0132 | T-109 | “ | RSV  8EC50 (μM) | DPLVFPSDEFDASISQVNEKINQSLAFIRKSDELL=35 | “ |
| AVP0133 | T-110 | “ | RSV  30EC50 (μM) | PLVFPSDEFDASISQVNEKINQSLAFIRKSDELLH=35 | “ |
| AVP0134 | T-111 | “ | RSV  9EC50 (μM) | LVFPSDEFDASISQVNEKINQSLAFIRKSDELLHN=35 | “ |
| AVP0135 | T-112 | “ | RSV  19EC50 (μM) | VFPSDEFDASISQVNEKINQSLAFIRKSDELLHNV=35 | “ |
| AVP0136 | T-113 | “ | RSV  8EC50 (μM) | FPSDEFDASISQVNEKINQSLAFIRKSDELLHNVN=35 | “ |
| AVP0137 | T-114 | “ | RSV6EC50 (μM) | PSDEFDASISQVNEKINQSLAFIRKSDELLHNVNA=35 | “ |
| AVP0138 | T-115 | “ | RSV6EC50 (μM) | SDEFDASISQVNEKINQSLAFIRKSDELLHNVNAG=35 | “ |
| AVP0139 | T-116 | “ | RSV12EC50 (μM) | DEFDASISQVNEKINQSLAFIRKSDELLHNVNAGK=35 | “ |
| AVP0140 | T-117 | “ | RSV13EC50 (μM) | EFDASISQVNEKINQSLAFIRKSDELLHNVNAGKS=35 | “ |
| AVP0141 | T-118 | “ | RSV  6EC50 (μM) | FDASISQVNEKINQSLAFIRKSDELLHNVNAGKST=35 | “ |
| AVP0142 | T-119 | “ | RSV  8EC50 (μM) | DASISQVNEKINQSLAFIRKSDELLHNVNAGKSTT=35 | “ |
| AVP0143 | T-118 | “ | RSV0.051EC50 (μM) | FDASISQVNEKINQSLAFIRKSDELLHNVNAGKST=35 | “ |
| AVP0427 | Peptide 80 to 94 | RSV Rho-A protein | RSV  1.6μg/ml[Virus entry](http://crdd.osdd.net/servers/avpdb/browse.php?by=Virus%20entry&TYPE=Target) | ILMCFSIDSPDSLEN=15 | Budge PJ, Li Y, Beeler JA, Graham BS. RhoA-derived peptide dimers share mechanistic properties with other polyanionic inhibitors of respiratory syncytial virus (RSV), including disruption of viral attachment and dependence on RSV G. J Virol. 2004;78(10):5015-22. |
| AVP0428 | Peptide 83A | RSV Rho-A protein | RSV>100μg/ml | ILMAFSIDSPDSLEN=15 | “ |
| AVP0429 | Peptide 80 to 94-N | RSV Rho-A protein | RSV>100μg/ml | ILMCFSINSPNSLQN=15 | “ |
| AVP1310 | T-142 | RSV fusion (F) protein | RSV IC50=Medium  [Fusion](http://crdd.osdd.net/servers/avpdb/browse.php?by=Fusion&TYPE=Target) | YTSVITIELSNIKENKCNGTDAKVKLIKQELDKYK=35 | Dani Paul Bolognesi, Thomas James Matthews, Carl T. Wild, Shawn O'Lin Barney,Dennis Michael Lambert, Stephen Robert Petteway, 1999: Methods for the inhibition of respiratory syncytial virus transmission. Trimeris, Inc., Durham NC. J. Enz. Inhib(6):99-111 |
| AVP1311 | T-143 | “ | RSV  High | TSVITIELSNIKENKCNGTDAKVKLIKQELDKYKN=35 | “ |
| AVP1312 | T-144 | “ | RSV  Nil | SVITIELSNIKENKCNGTDAKVKLIKQELDKYKNA=35 | “ |
| AVP1313 | T-145 | “ | “ | VITIELSNIKENKCNGTDAKVKLIKQELDKYKNAV=35 | “ |
| AVP1314 | T-146 | “ | “ | ITIELSNIKENKCNGTDAKVKLIKQELDKYKNAVT=35 | “ |
| AVP1315 | T-147 | “ | “ | TIELSNIKENKCNGTDAKVKLIKQELDKYKNAVTE=35 | “ |
| AVP1316 | T-148 | “ | “ | IELSNIKENKCNGTDAKVKLIKQELDKYKNAVTEL=35 | “ |
| AVP1317 | T-149 | “ | “ | ELSNIKENKCNGTDAKVKLIKQELDKYKNAVTELQ=35 | “ |
| AVP1318 | T-150 | RSV fusion (F) protein | “ | LSNIKENKCNGTDAKVKLIKQELDKYKNAVTELQL=35 | “ |
| AVP1319 | T-151 | “ | “ | SNIKENKCNGTDAKVKLIKQELDKYKNAVTELQLL=35 | “ |
| AVP1320 | T-152 | “ | “ | NIKENKCNGTDAKVKLIKQELDKYKNAVTELQLLM=35 | “ |
| AVP1321 | T-153 | “ | “ | IKENKCNGTDAKVKLIKQELDKYKNAVTELQLLMQ=35 | “ |
| AVP1322 | T-154 | “ | “ | KENKCNGTDAKVKLIKQELDKYKNAVTELQLLMQS=35 | “ |
| AVP1323 | T-155 | “ | “ | ENKCNGTDAKVKLIKQELDKYKNAVTELQLLMQST=35 | “ |
| AVP1324 | T-104 | “ | RSVLow | IINFYDPLVFPSDEFDASISQVNEKINQSLAFIRK=35 | “ |
| AVP1325 | T-105 | **“** | RSV**Inhibition/IC50** Low | INFYDPLVFPSDEFDASISQVNEKINQSLAFIRKS=35 | “ |
| AVP1326 | T-106 | “ | RSV **Inhibition/IC50** Low | NFYDPLVFPSDEFDASISQVNEKINQSLAFIRKSD=35 | “ |
| AVP1327 | T-107 | “ | RSVMedium | FYDPLVFPSDEFDASISQVNEKINQSLAFIRKSDE=35 | “ |
| AVP1328 | T-108 | “ | RSVHigh | YDPLVFPSDEFDASISQVNEKINQSLAFIRKSDEL=35 | “ |
| AVP1329 | T-109 | “ | “ | DPLVFPSDEFDASISQVNEKINQSLAFIRKSDELL=35 | “ |
| AVP1330 | T-110 | “ | RSVMedium | PLVFPSDEFDASISQVNEKINQSLAFIRKSDELLH=35 | “ |
| AVP1331 | T-111 | “ | RSVHigh | LVFPSDEFDASISQVNEKINQSLAFIRKSDELLHN=35 | “ |
| AVP1332 | T-112 | “ | **“ Inhibition/IC50** High | VFPSDEFDASISQVNEKINQSLAFIRKSDELLHNV=35 | “ |
| AVP1333 | T-113 | “ | “ | FPSDEFDASISQVNEKINQSLAFIRKSDELLHNVN=35 | “ |
| AVP1334 | T-114 | “ | “ | PSDEFDASISQVNEKINQSLAFIRKSDELLHNVNA=35 | “ |
| AVP1335 | T-115 | “ | “ | SDEFDASISQVNEKINQSLAFIRKSDELLHNVNAG=35 | “ |
| AVP1336 | T-116 | “ | “ | DEFDASISQVNEKINQSLAFIRKSDELLHNVNAGK=35 | “ |
| AVP1337 | T-117 | “ | “ | EFDASISQVNEKINQSLAFIRKSDELLHNVNAGKS=35 | “ |
| AVP1338 | T-118 | “ | “ | FDASISQVNEKINQSLAFIRKSDELLHNVNAGKSI=35 | “ |
| AVP1924 | 77-95 | Rho-A protein | RSV  IC50=7.6μM[Replication](http://crdd.osdd.net/servers/avpdb/browse.php?by=Replication&TYPE=Target) | TDVILMCFSIDSPDSLENI=19 | Budge PJ, Lebowitz J, Graham BS.2003; Antiviral activity of RhoA-derived peptides against respiratory syncytial virus is dependent on formation of peptide dimers.Antimicrob Agents Chemother. 47(11):3470-7. |
| [AVP1925](http://crdd.osdd.net/servers/avpdb/record.php?details=AVP1925) | 77-95 | “ | RSV>50μM | CSIELSDIPLSVDFNTMID=19 | “ |
| [AVP1926](http://crdd.osdd.net/servers/avpdb/record.php?details=AVP1926) | 77-95-77A | “ | RSV2.56μM | ADVILMCFSIDSPDSLENI=19 | “ |
| AVP1927 | 77-95-78A | “ | RSV1.37μM | TAVILMCFSIDSPDSLENI=19 | “ |
| AVP1928 | 77-95-79A | “ | RSV6.6μM | TDAILMCFSIDSPDSLENI=19 | “ |
| AVP1929 | 77-95-80A | “ | RSV11.6μM | TDVALMCFSIDSPDSLENI=19 | “ |
| AVP1930 | 77-95-81A | “ | RSV5.42μM | TDVIAMCFSIDSPDSLENI=19 | “ |
| AVP1931 | 77-95-82A | “ | RSV1.43μM | TDVILACFSIDSPDSLENI=19 | “ |
| AVP1932 | 77-95-83A | “ | RSV>50μM | TDVILMAFSIDSPDSLENI=19 | “ |
| AVP1933 | 77-95-84A | “ | RSV6.29μM | TDVILMCASIDSPDSLENI=19 | “ |
| AVP1934 | 77-95-85A | “ | RSV6.82μM | TDVILMCFAIDSPDSLENI=19 | “ |
| AVP1935 | 77-95-86A | “ | RSV3.52μM | TDVILMCFSADSPDSLENI=19 | “ |
| AVP1936 | 77-95-87A | “ | RSV4.36μM | TDVILMCFSIASPDSLENI=19 | “ |
| AVP1937 | 77-95-88A | “ | RSV2.26μM | TDVILMCFSIDAPDSLENI=19 | “ |
| AVP1938 | 77-95-89A | “ | RSV15.32μM | TDVILMCFSIDSADSLENI=19 | “ |
| AVP1939 | 77-95-90A | “ | RSV2.61μM | TDVILMCFSIDSPASLENI=19 | “ |
| AVP1940 | 77-95-91A | “ | RSV1.19μM | TDVILMCFSIDSPDALENI=19 | “ |
| AVP1941 | 77-95-92A | “ | RSV2.27μM | TDVILMCFSIDSPDSAENI=19 | “ |
| AVP1942 | 77-95-93A | “ | RSV9.83μM | TDVILMCFSIDSPDSLANI=19 | “ |
| AVP1943 | 77-95-94A | “ | RSV18.47μM | TDVILMCFSIDSPDSLEAI=19 | “ |
| AVP1944 | 77-95-95A | “ | RSV4.89μM | TDVILMCFSIDSPDSLENA=19 | “ |
| AVP1945 | 77-86 | “ | RSV>50μM | TDVILMCFSI=10 | “ |
| AVP1946 | 77�89 | “ | RSV>50μM | TDVILMCFSIDSP=13 | “ |
| AVP1947 | 77-92 | “ | RSV10.86μM | TDVILMCFSIDSPDSL=16 | “ |
| AVP1948 | 78-95 | “ | RSV1.23μM | DVILMCFSIDSPDSLENI=18 | “ |
| AVP1949 | 79-95 | “ | RSV16.95μM | VILMCFSIDSPDSLENI=17 | “ |
| AVP1950 | 80-95 | “ | RSV7.17μM | ILMCFSIDSPDSLENI=16 | “ |
| AVP1951 | 83-95 | “ | RSV>50μM | CFSIDSPDSLENI=15 | “ |
| AVP1952 | 80-94 | “ | RSV1.75μM | ILMCFSIDSPDSLEN=15 | “ |
| AVP1953 | 80-93 | “ | RSV3.5μM | ILMCFSIDSPDSLE=14 | “ |
| AVP1954 | 80-92 | “ | RSV12.4μM | ILMCFSIDSPDSL=13 | “ |
| AVP1955 | 80-91 | “ | RSV6.36μM | ILMCFSIDSPDS=12 | “ |
| AVP1956 | 80-90 | “ | RSV4.61μM | ILMCFSIDSPD=11 | “ |
| AVP1957 | 80-89 | “ | RSV35.77μM | ILMCFSIDSP=10 | “ |
| AVP1958 | 80-88 | “ | RSV>50μM | ILMCFSIDS=9 | “ |
| AVP1959 | 80-87 | “ | RSV>50μM | ILMCFSID=8 | “ |
| AVP1960 | 80-86 | “ | RSV>50μM | ILMCFSI=7 | “ |
| AVP1961 | 80-85 | “ | RSV>50μM | ILMCFS=6 | “ |
| AVP1971 | HR1-30a | RSV fusion (F) protein | RSV1.68μM fusion | AVSKVLHLEGEVNKISALLSTNKAVVSLSNGVSVLTSKVLDLDNYIDKQLLPIVNK=20 | Wang E, Sun X, Qian Y, Zhao L, Tien P, Gao GF. Both heptad repeats of human respiratory syncytial virus fusion protein are potent inhibitors of viral fusion.Biochem Biophys Res Commun. 2003 :14;302(3):469-75 |
| AVP1972 | HR2-30a | RSV fusion (F) protein | RSV2.93μM[Fusion](http://crdd.osdd.net/servers/avpdb/browse.php?by=Fusion&TYPE=Target) | NFYDPLVFPSDEFDASISQVNEKINQSLASIRKSDELLHNVNAGK=45 | “ |
| AVP2000 | G149-197 | RSV attachment glycoprotein | RSV80μM[Cytopathic effect](http://crdd.osdd.net/servers/avpdb/browse.php?by=Cytopathic%20effect&TYPE=Target) | KQRQNKPPSKPNNDFHFEVFNFVPCSICSNNPTCWAICKRIPNKKPGKK=49 | Gorman JJ, McKimm-Breschkin JL, Norton RS, Barnham KJ.Antiviral activity and structural characteristics of the nonglycosylated central subdomain of human respiratory syncytial virus attachment (G) glycoprotein.[J Biol Chem.](https://www.ncbi.nlm.nih.gov/pubmed/11487583) 2001 Oct 19;276(42):38988-94 |
| AVP2001 | G163-197 | “ | “ | FHFEVFNFVPCSICSNNPTCWAICKRIPNKKPGKK=35 | “ |
| AVP2002 | G171-197 | “ | RSV>165μM | VPCSICSNNPTCWAICKRIPNKKPGKK=27 | “ |
| AVP2003 | G173-197 | “ | RSV>177μM | CSICSNNPTCWAICKRIPNKKPGKK=25 | “ |
| AVP2004 | G149-189 | “ | RSV12μM | KQRQNKPPSKPNNDFHFEVFNFVPCSICSNNPTCWAICKRI=41 | “ |
| AVP2005 | G154-189 | “ | RSV12μM | KPPSKPNNDFHFEVFNFVPCSICSNNPTCWAICKRI=36 | “ |
| AVP2006 | G158-189 | “ | RSV25μM | KPNNDFHFEVFNFVPCSICSNNPTCWAICKRI=32 | “ |
| AVP2007 | G149-169 | “ | RSV>190μM | KQRQNKPPSKPNNDFHFEVFN=21 | “ |
| AVP2008 | G149-165 | “ | RSV>240μM | KQRQNKPPSKPNNDFHF=17 | “ |
| AVP2009 | G154-172 | “ | RSV220μM | KPPSKPNNDFHFEVFNFVP=19 | “ |
| AVP2010 | G154-171 | “ | RSV14μM | KPPSKPNNDFHFEVFNFV=18 | “ |
| AVP2011 | G154-170 | “ | RSV7μM | KPPSKPNNDFHFEVFNF=17 | “ |
| AVP2012 | G154-169 | “ | RSV>510μM | KPPSKPNNDFHFEVFN=16 | “ |
