# Supplementary material for: Building HMM and molecular docking analysis for the sensitive detection of anti-viral pneumonia antimicrobial peptides (AMPs)
Source: Sci Rep. 2021 Oct 18;11:20621. doi: 10.1038/s41598-021-00223-8 (PMC8523717; doi:10.1038/s41598-021-00223-8)
Supplement: Supplementary file 1 — Supplementary Information. [file 41598_2021_223_MOESM1_ESM.zip › Supplemetary final/Novel putative Antiviral peptides.docx]

INFLUENZA A

1. TPTFIDGQVPIPKQ
2. CPVILDSAIQVLPK
3. TPTFIDGQVTVPDQ
4. TPTFIDGQVPMPQQ
5. TPTFIDGQVPIPQQ
6. TPKFIDGQVPIPEQ
7. CPVILDSSIQVFPK
8. TPTFIDGQVPIPEQ

INFLUENZA B

1. MDVSHRWTFLRVPPQ
2. MDVSHRWTFLMVPPQ
3. LNCNPQLLCLNCNPQ
4. LKILQLLLFLKVPQL
5. LTILQLLLFLKVPQL
6. LNCNPPLLCLNCNPQ

RESPIRATORY SYNCYTIAL VIRUS

1. IVSSIKEEINLCKNKF
2. EVSKINEKIDSNLSTV
3. NIVDVNKKIDANTTAI
4. KVTEINANIDNNVNII
5. AINSVSAQVNKNTNNI
6. KFSQICEAIDMQTSVI
7. NISNVKNELNQNINNV
8. SIYNFNENSNVLLSAV
9. NISKVTNQVNTNTTNI
10. VINDVSKQVNTNTTNI
11. NVTNISNKVDVNTADI
12. DITNINNNIDAKFTKI
13. KIRDLNEKLDDRITNV
